# iSubgraph: Integrative Genomics for Subgroup Discovery in Hepatocellular Carcinoma Using Graph Mining and Mixture Models

**DOI:** 10.1371/journal.pone.0078624

**Published:** 2013-11-04

**Authors:** Bahadir Ozdemir, Wael Abd-Almageed, Stephanie Roessler, Xin Wei Wang

**Affiliations:** 1 Department of Computer Science, University of Maryland, College Park, Maryland, United States of America; 2 Liver Carcinogenesis Section, Laboratory of Human Carcinogenesis, Center for Cancer Research, National Cancer Institute, Bethesda, Maryland, United States of America; 3 Institute for Advanced Computer Studies, University of Maryland, College Park, Maryland, United States of America; 4 Institute of Pathology, Heidelberg University Hospital, Heidelberg, Germany; Icahn School of Medicine at Mount Sinai, United States of America

## Abstract

The high tumor heterogeneity makes it very challenging to identify key tumorigenic pathways as therapeutic targets. The integration of multiple omics data is a promising approach to identify driving regulatory networks in patient subgroups. Here, we propose a novel conceptual framework to discover patterns of miRNA-gene networks, observed frequently up- or down-regulated in a group of patients and to use such networks for patient stratification in hepatocellular carcinoma (HCC). We developed an integrative subgraph mining approach, called iSubgraph, and identified altered regulatory networks frequently observed in HCC patients. The miRNA and gene expression profiles were jointly analyzed in a graph structure. We defined a method to transform microarray data into graph representation that encodes miRNA and gene expression levels and the interactions between them as well. The iSubgraph algorithm was capable to detect cooperative regulation of miRNAs and genes even if it occurred only in some patients. Next, the miRNA-mRNA modules were used in an unsupervised class prediction model to discover HCC subgroups via patient clustering by mixture models. The robustness analysis of the mixture model showed that the class predictions are highly stable. Moreover, the Kaplan-Meier survival analysis revealed that the HCC subgroups identified by the algorithm have different survival characteristics. The pathway analyses of the miRNA-mRNA co-modules identified by the algorithm demonstrate key roles of Myc, E2F1, let-7, TGFB1, TNF and EGFR in HCC subgroups. Thus, our method can integrate various omics data derived from different platforms and with different dynamic scales to better define molecular tumor subtypes. iSubgraph is available as MATLAB code at http://www.cs.umd.edu/~ozdemir/isubgraph/.

## Introduction

Scientists have made great progress in the development of new treatment modalities for certain cancer types over the past three decades. However, the improvement of mortality rates in cancer patients remains very modest, especially for esophageal, liver, lung and pancreatic cancers [1]. Tumor heterogeneity is the major obstacle that we need to overcome in order to improve cancer treatment outcomes and patient mortality rates. Similar to other lethal tumors, most primary liver cancer patients cannot be cured because of extensive tumor heterogeneity. Liver cancer represents a heterogeneous group of malignancies attributable to a variety of genetic and environmental causes, such as different cells of origin, range in patient ethnicity, etiology, underlying disease and diversity of genomic and epigenomic changes which drive tumor development [2].

Recent advances in molecular-based technologies have enabled researchers to recognize molecular differences between tumors from different patients, inter-tumor heterogeneity, and between different areas of an individual tumor, intra-tumor heterogeneity, possibly originating from the existence of cancer stem cells or selection by clonal evolution. The high degree of heterogeneity observed in the hepatocellular carcinoma (HCC) population implies that multiple patient subgroups exist, each of which share similar tumor biology [3]. Molecularly targeted therapies are promising new treatment options because they are highly effective in a stratified group of patients. Therefore, even though they may not fundamentally reduce overall mortality in the whole cohort, selection of patients that may respond to a specific treatment might lead to greatly improved outcome in this subgroup. Thus, our ability to identify distinct groups of cancer patients with similar tumor biology who are most likely to respond to a specific therapy would have a significant impact on improving patient outcome.

MicroRNAs (miRNAs) are 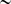
22 nucleotide long non-coding RNAs that take a significant role in regulation of gene networks by targeting complementary messenger RNA (mRNA) transcripts [4], [5]. Previous miRNA expression profiling studies have identified a few differentially expressed miRNAs in liver tumor such as miR-122, miR-26, and miR-101, which are down-regulated in HCC, and miR-21 and miR-221, which are up-regulated in HCC [6], [7]. However, the functions of miRNAs in complex cellular systems have not yet been fully understood because accurate prediction of post-transcriptional gene regulatory mechanisms poses a major challenge for most miRNAs.

Changes in both miRNA and gene expression levels observed specifically in a subgroup of cancer patients might be the result of driving regulatory pathways between miRNAs and genes. For example, an abundant miRNA can repress the translation of its target genes, which may lead to the down-regulation of hundreds of genes. Most prognostic signatures that have been proposed to predict clinical outcome for HCC are based on either miRNA or gene expression profiles. Such signatures can identify only one side of the signaling pathway. On the other hand, integrated analysis of miRNA and gene expression data offers a broader view of the big picture and may help to develop more accurate prediction models.

Systems biology is a holistic approach that strives the integration of experimental information into computational models that contribute towards further understanding of complex biological systems [8]–[10]. The goal of this study was to provide a conceptual framework that allows integration of different genomic data modalities and their interactions into graphical network models in order to better understand biological pathways associated with a specific type of cancer and to identify related molecular networks. We specifically focused on the integration of miRNA and mRNA expression profiles for liver cancer. However, the method we propose is easily extensible to other omics data types such as DNA methylation data, somatic copy number alterations or metabolomics. Therefore, systems biology approaches, such as integrating miRNA and gene expression profiles, may aid in the understanding of their interaction in liver tumor cells and their contribution to the pathogenesis of liver cancer.

We propose a simple yet powerful tool to discover patterns of miRNAs and their target genes, observed frequently up- or down-regulated in a group of patients. Our method is capable to detect cooperative regulation of miRNAs and genes even if it occurs in some patients only. We denoted each patient by a single bipartite graph, which encodes miRNA and gene expression levels and also the interactions between them. We used an integrative graph mining approach called iSubgraph (integrated subgraph analysis) to identify miRNA-mRNA regulatory networks frequently seen in HCC as subgraphs. These miRNA-mRNA modules were used in a stable unsupervised class prediction model for patient stratification to discover HCC subgroups. The Kaplan-Meier survival analysis showed that subgroups identified by the algorithm have different survival characteristics. The statistical analyses of the biologically significant miRNA-mRNA co-modules identified by the algorithm demonstrate key roles of Myc, E2F1, let-7, TGFB1, TNF and EGFR in HCC subgroups. Thus, this approach can integrate omics data with different dynamic scales to better define tumor subgroups.

## Materials and Methods

### Study Cohorts

In this study, we used three HCC cohorts with publicly available microarray data at the Gene Expression Omnibus (GEO; http://www.ncbi.nlm.nih.gov/geo) database. The gene and miRNA expression data of the first cohort have been published earlier in [11] and [12], respectively. The microarray data accession numbers are GSE14520 and GSE6857 for the gene and miRNA expression. This cohort consists of 242 HCC cases derived from Liver Cancer Institute (LCI) of Fudan University. The second cohort is composed of HCC patients who received primary curative hepatectomy at Queen Mary Hospital (Pokfulam, Hong Kong) between 1990 and 2007. The expression data of this cohort have been studied in [13] and [14]. For the Hong Kong cohort, the gene and miRNA expression profiles from 96 tumor and adjacent nontumor tissues are available under accession number GSE22058. The gene expression profiling of the third cohort was performed by the Laboratory of Experimental Carcinogenesis (LEC, National Cancer Institute) and the data has been published previously in [15] and [16]. The gene expression of the LEC cohort data can be accessed at GEO with accession numbers GSE1898 and GSE4024. Only gene expression data is available for this cohort. Therefore, we used this dataset for validation purposes only. The LEC cohort consists of 139 HCC patients and the microarray platform contains 16,796 genes. For 113 patients, disease free survival and overall survival were available. In the experiments, we used only the patients with survival data. Significant amount of the expression data is missing for this dataset (32% of the data). The Bayesian mixture model, used for clustering, can resolve this issue by marginalizing probabilities over missing values. For all cohorts, pre-normalized data as published previously was used for statistical analyses in logarithmic scale. The mRNA data of the LCI cohort was RMA normalized and the miRNA data was median and to an artificial common reference normalized. Of the Hong Kong the mRNA data was RMA normalized and the log10 expression level of the miRNA data (qPCR data) was normalized to the sample mean. For the LEC mRNA data the log2 transformed intensity ratios of each spot from duplicated experiments were median normalized. The patient characteristics for the LCI and LEC cohorts are shown in Table 1. The LCI and LEC cohorts of this study were the subsets of those used in [11] since we removed the patients for whom we did not have miRNA, gene expression and survival data.

**Table 1:** Clinical characteristics of patients in the LCI and LEC cohorts at the time of surgery.

Clinical variable	LCI Cohort ( 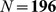 )	LEC Cohort ( 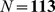 )	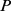 -value^a^
AVR-CC (yes/no/NA)	54/136/6	NA	NA
Gender (male/female/NA)	171/25/0	81/32/0	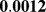
Age ( 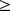 50 y/<50 y/NA)	108/88/0	70/43/0	0.2822
AFP (>300 ng/mL/ 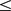 300 ng/mL/NA)	86/106/0	51/53/9	0.5419
ALT (>50 U/L/ 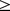 50 U/L/NA)	79/117/0	NA	NA
Cirrhosis (yes/no/NA)	181/15/0	49/64/0	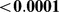
Tumor size (>5 cm/ 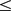 5 cm/NA)	72/123/1	65/48/0	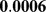
Multinodular (yes/no/NA)	41/155/0	NA	NA
BCLC staging (B–C/A-0/NA)	46/149/1	NA	NA
CLIP staging (1–5/0/NA)	108/87/1	NA	NA
TNM staging (II–III/I/NA)	114/81/1	NA	NA
Survival at 60 mo (events/censored/NA)	77/119/0	67/46/0	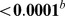

Abbreviations: NA, not available; available; AVR-CC, active viral replication chronic carrier.

^*a*^Fisher' sexacttest;

^*b*^Cox-Mantel log-rank test.

### Data Preprocessing

We combined the probes that matched to the same miRNAs listed in the miRBase database (release 19, http://www.mirbase.org) [17] using their median expression level. Probes that did not match to any miRNA were removed from the dataset. The final microarray data of the LCI cohort had 196 tumor and 185 adjacent nontumor samples from 196 patients with 13,101 genes and 206 miRNAs with missing values for some cases (7% of the data). The same procedure for combining miRNA probes was applied on the Hong Kong cohort which resulted in 18,503 genes and 202 miRNAs.

### Statistical Analyses and Computational Methods

The robustness of a clustering algorithm is a crucial issue in tumor subclassification. An unstable clustering method might yield quite distinct patient subgroups when it is applied on different cohorts. The problem of unstable class estimates may stem from over fitting of the model to training data due to very high dimensionality of microarray data with limited number of samples. Therefore, one can take feature selection approaches to prevent the curse of dimensionality [18] by narrowing down the set of genes and miRNAs. In addition, the complexity of the clustering method i.e. the number of free parameters can be reduced to avoid over fitting. Thus, we used a stable clustering method for cancer patients by taking both approaches as described below.

The iSubgraph algorithm first discovers regulatory networks of miRNAs and their target genes, which are frequently observed in a subset of patients as up- or down-regulated. Next, it uses the expression levels of only those genes and miRNAs in an unsupervised clustering method to discover cancer subgroups. The iSubgraph algorithm can be briefly summarized in three main parts.

1. Transforming microarray data into graph representation,
2. Mining frequent subgraphs,
3. Cancer subgroup discovery via clustering using mixture models.

**Figure 1:**
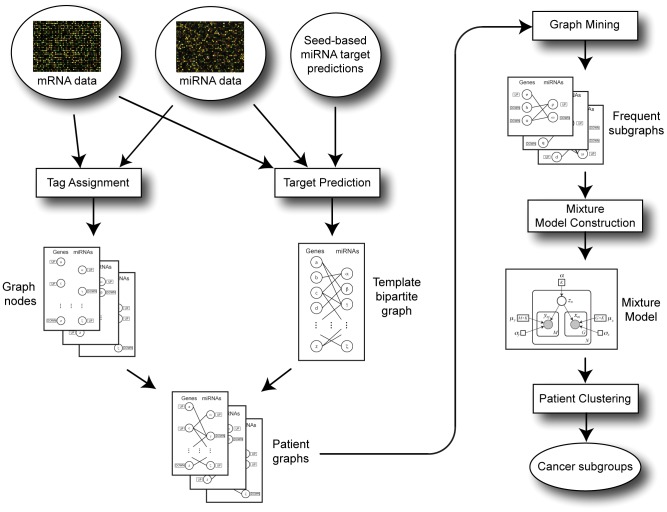
Schematic overview of the iSubgraph algorithm. The flow chart illustrates the transformation of microarray data into graph representation (left); followed by the graph mining-based method to identify significant miRNA-gene co-modules (top right) and tumor subclassification by the mixture model (bottom right).

Figure 1 shows the flow chart of the overall algorithm. The graph representation of patients serves as an abstraction of microarray data, which enables making generalization about patients that belong to the same cancer subgroup. Instead of using exact expression levels of genes and miRNAs, these values were discretized, for generalization, into three groups as over-expressed, under-expressed or averagely-expressed. Similarly, instead of quantification of miRNA-gene interactions, we showed these targeting interactions in graphs by three possible relationship types: positively correlated, negatively correlated, or no target relationship. The graph construction starts with two parallel steps: target gene prediction for miRNAs and identification of differentially expressed cases of each gene and each miRNA. The former step specifies a template bipartite graph where miRNA-mRNA targeting relationships are represented by edges, while the latter provides graph nodes for genes and miRNAs with appropriate tags (UP or DOWN) for each tumor sample. In the next step, these edges and nodes are merged into bipartite graphs called patient graphs.

The details of graph construction are described as follows: Target genes of miRNAs were determined by filtering the predictions of the TargetScan algorithm [19], which identifies mRNAs with conserved complementarity to the miRNA seed. Target predictions were tested for correlation (or anti-correlation) between miRNA and gene expression levels of both tumor and adjacent nontumor tissues. Significantly correlated pairs were considered as actual miRNA-mRNA interactions. Let vectors 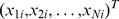 and 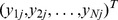 be the logarithms of the expression levels of the 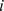
th gene and the 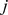
th miRNA, in samples 1 to 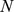 from both tumor and nontumor tissues, respectively. Suppose that the 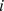
th gene is predicted as a potential target of the 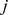
th miRNA in the TargetScan database. We first compute the correlation coefficient, 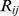, between these expression vectors. The type of targeting relationship is determined from the sign of 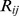 (positively or negatively correlated). Then, the significance of Pearson correlation (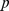-value) is assessed by one-tailed test. This includes permuting the expression values in each vector 1000 times, recalculating correlation coefficient for each permutation, and counting significant results. Finally, multiple comparisons procedure was performed for the targets of each miRNA to control false discovery rate of the genes reported by the source of target prediction knowledge (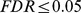). The pseudocode of the target prediction procedure is shown in Procedure S1. We used a bipartite graph, which represents miRNA-gene interactions, as a template for constructing patient graphs. In this graph, two disjoint vertex sets represent genes and miRNAs in the dataset where each gene and each miRNA correspond a certain vertex in the graph. A miRNA vertex and a gene vertex were connected by an edge if there exists a positively or negatively correlated target relationship between the corresponding gene and miRNA.

The next step of the method was to construct one bipartite graph for each patient based on the template bipartite graph. Patient graphs, constructed from tumor samples, contained only up- or down-regulated genes and miRNAs in those patients. The objective here is to create a set of structured data models that facilitate the detection of abnormalities in the expression profiles of a cancer subgroup. A patient graph had a graph node attached with UP or DOWN tag if the corresponding gene or miRNA was over-expressed or under-expressed in that patient, respectively. Defining cutoff values for over- and under-expressed genes/miRNAs can solve such a tag assignment problem. The nodes without a tag, which indicated they were neither up- nor down-regulated in that patient, were removed from patient graphs. The edges between the remaining nodes were also removed if the correlation type of an edge was not in accordance with the tags of the nodes that were incident to that edge. For example, an edge between an up-regulated miRNA node and an up-regulated gene node was removed from a patient graph if they were negatively correlated in the template bipartite graph. After this process was repeated for all genes and miRNAs, we obtained 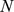 different bipartite graphs representing 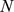 patients with their up- or down-regulated genes and miRNAs. Moreover, these graphs encoded the interactions between genes and miRNAs. The pseudocode of the graph construction part is provided in Procedure S2.

The second part of the iSubgraph algorithm is to mine frequent cooperative regulation of genes and miRNAs in tumor samples. Such regulation mechanisms used by tumor cells can increase or decrease specific gene products in some cancer patients. Considering the patient graphs constructed in the first part, we can discern similar patterns of connected graph nodes with UP or DOWN tags in some patients as the results of such mechanisms. In other words, a miRNA-driven gene regulatory network corresponds to a subset of nodes and edges of a patient graph, i.e. a subgraph. Since we had a bipartite graph for each patient, we were able to identify frequently observed regulatory miRNA-mRNA networks using graph mining algorithms. These algorithms aim to extract useful information from structured datasets by finding interesting graph patterns that might be frequent, correlated or discriminative in a set of graphs. Before explaining how to retrieve such networks, we provide some preliminary concepts about graph mining below:

#### Definition 1 (Labeled Graph)

*A labeled graph has labels assigned to its vertices and edges. It can be represented by 4-tuple, G* = (*V*, *E*, *L*, 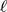) *where V is a set of vertices,* 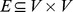 *is a set of edges, L is a set of labels and* 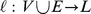 *is a function that maps a vertex or an edge to a label.*

In our case, the label of a vertex was either UP or DOWN, whereas all edges were labeled with empty labels.

We say that a graph 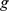 is a subgraph of a graph 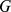 if the vertex set of 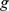 is a subset of that of 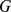, and the edge set of 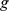 is a subset of that of 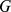. This relationship is denoted by 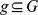 (and 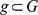 if proper subgraph). In the opposite direction, we say that a graph 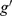 is a supergraph of a graph 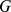 if 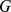 is a subgraph of 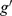.

#### Definition 2 (Subgraph isomorphism)

*Let* 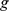 *and* 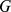 *be two labeled graphs, then a subgraph isomorphism of* 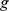 *to* 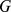 *is an injective function* 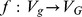, *that satisfies* 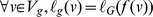; *also* 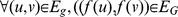 *and* 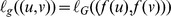.

One can say that a graph 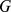 contains another graph 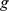 if there exists a subgraph of 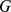 to which 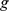 is isomorphic.

#### Definition 3 (Frequent subgraph mining:[20])

*Given a graph dataset*, 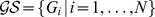, *and a minimum support*, 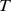; *let*

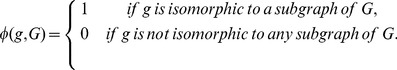
(1) and
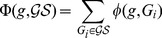
(2)
*where* 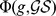 *denotes the occurrence frequency of* 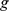 *in* 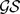, *i.e., the support of* 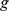 *in* 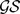. *Frequent subgraph mining is defined as finding every graph* 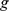, *such that* 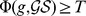.

Graph mining algorithms are inherently based on search algorithms such as breadth-first search where the state space consists of potential frequent subgraphs. The search for all frequent subgraphs starts with an empty subgraph. Either a node or an edge is added to the candidate subgraph at every level as the search deepens. The new candidate subgraph is inserted to the set of frequent subgraphs if its support is equal or greater than the frequency threshold; otherwise the search is pruned on that subtree. An overview of several methods and related problems can be found in [21]. In our problem formulation, regulatory miRNA-mRNA networks matched with frequent subgraphs and their supports were equal to the number of patients that possess such co-regulation patterns. The set of all frequent subgraphs were highly redundant because most of them shared the same gene and miRNA nodes. Therefore, we used only connected and closed frequent subgraphs in order to reduce redundancy without losing any information. A frequent subgraph 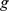 is *closed* if there is no proper supergraph of 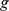 with the same support in the dataset. Let the set of frequent graph patterns, 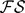, include all the graphs whose support is greater than or equal to a minimum support threshold, 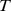; then the set of closed frequent graph patterns, 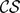, is defined as follows [20].
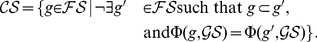
(3)


**Figure 2:**
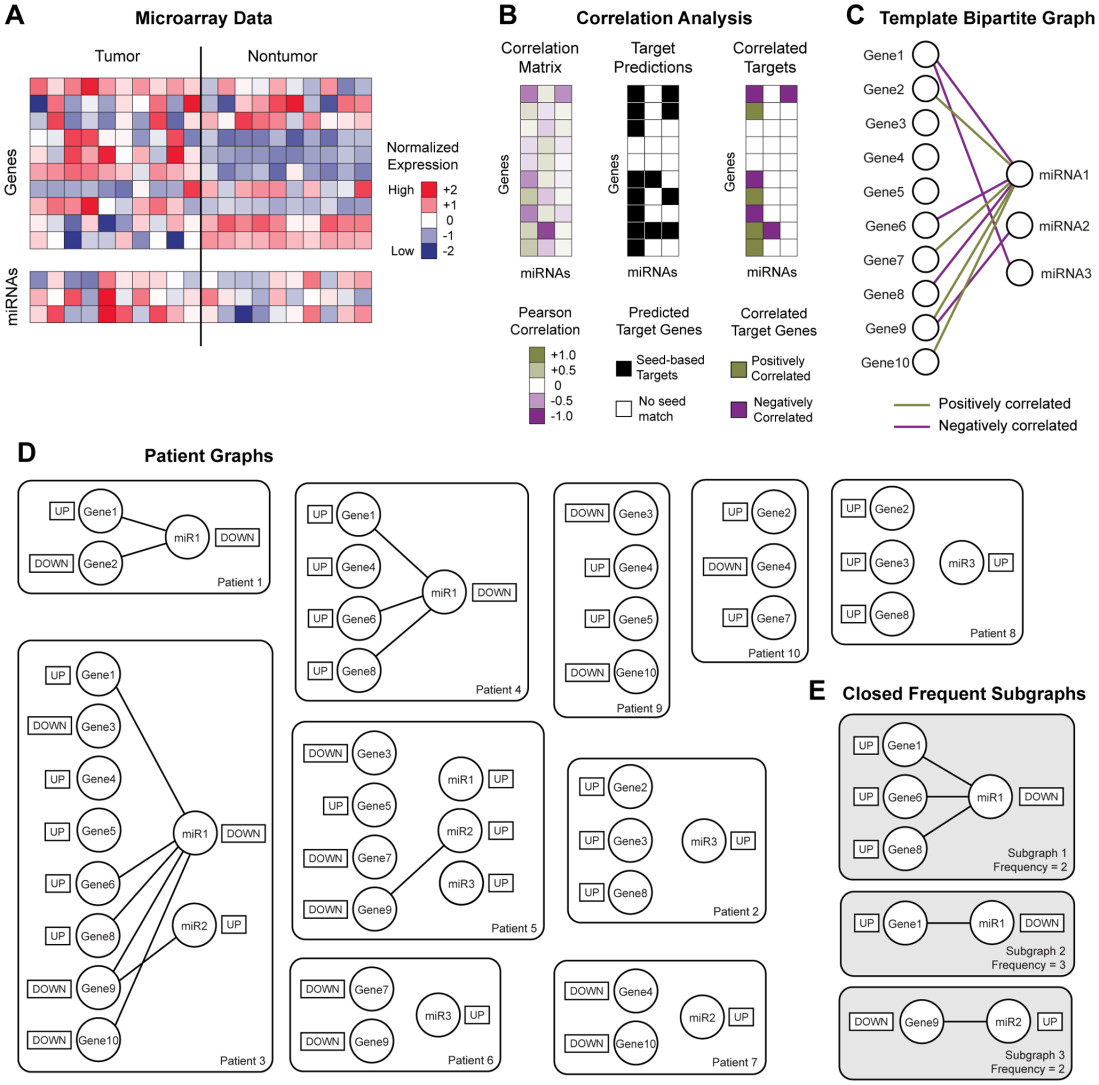
Graph Mining steps for a small dataset. (A) Normalized gene and miRNA expression levels in tumor and adjacent nontumor tissues. (B) Steps of correlation analysis to decide actual target genes of miRNAs. (C) Template bipartite graph representing miRNA-gene interactions. (D) Graph patients constructed for all patients. Threshold for UP and DOWN tags is 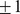. (E) All closed frequent subgraphs for a support threshold of 2 patients.

In addition to the minimum support threshold, we also defined a threshold for minimum number of gene nodes in frequent subgraphs so that we were able to remove subgraphs that were too specific to be informative about regulatory networks [22]. The graph mining procedure is illustrated in Figure 2 for a small sample dataset. For visualization purposes, we show only 10 patients where the support threshold is set to 2 patients.

The final filter on frequent subgraphs was about their statistical significance. Any frequent subgraph found by the graph mining algorithm with a reasonable support threshold is unlikely to be observed by chance. Nevertheless, we computed the statistical significance of every frequent subgraph by permuting sample labels independently for each gene and each miRNA (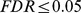).

Although mining only closed subgraphs significantly reduced the number of frequent subgraphs returned by the mining algorithm, they were still too redundant and required further processing. For example, two subgraphs differing in only one node would be reported individually if they had different supports. However, they were usually observed in the same patients and might have been associated with the same biological process. Therefore, merging all subgraphs into a single co-module aided eliminating the redundancy among subgraphs and provided a more generalized HCC-related regulatory network. Let 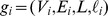 be the 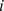 th subgraph in the set of closed frequent graph patterns, 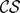; then the single co-module can be defined as 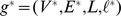 where 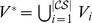 and 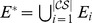. Similar to edges and vertices, subgraph labeling functions, 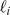, can be considered as a set of mapping rules from 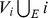 to 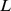, so the labeling function of the co-module can be formulated as union of mapping rules, 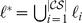.

**Figure 3:**
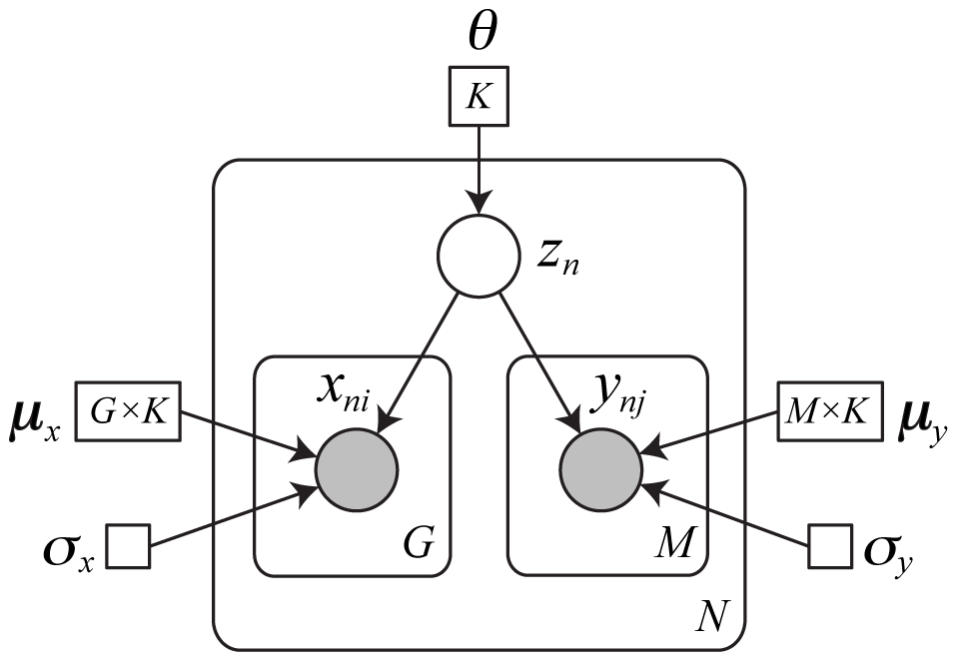
Mixture model for cancer subgroup discovery using plate notation. Circles indicate random variables: Subgroup of patient, 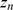, gene expression level, 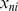, and miRNA expression level, 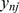. Shaded circles denote observed values. Outer rectangles indicate fixed paramters: Class mixing parameter, 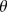, the mean expression level, 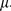, and shared standard deviation, 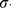. Directed edges show dependencies between variables and parameters. Capital letters represent the size of parameters (vector or matrix) and plate repetition.

The last part of the iSubgraph algorithm is cancer subgroup discovery via patient clustering. This stratification of patients may lead to the development of new targeted therapies. The patients in each cancer subgroup are expected to share similar tumor biology due to similar expression profiles. In the second part of the iSubgraph algorithm, we identified significant genes and miRNAs that play a role in HCC. The expression levels of all genes and miRNAs that occurred in frequent subgraphs were used as input into the subsequent clustering method. We employed a mixture model, which is a probabilistic model for representing subgroups with categorical latent variables, to cluster patients. The advantage of this model is to handle missing values easily. The model we used in the experiments is a version of the classical Gaussian mixture model [23]. Figure 3 demonstrates the graphical representation of this model. The model was trained from data using the expectation-maximization (EM) algorithm [23]. Although genes and miRNAs are functionally linked, the mixture model treats them independently because stable clustering of patients is superior to a more complex model. When clustering noisy data with EM, one can either define complex models or use robust estimation techniques [24]. On the other hand, we limited the genes and miRNAs used in the model to the ones found in frequent subgraphs so that the selected genes and miRNAs for the model were already considered as functionally relevant.

The EM algorithm for this model is explained as follows: In our mixture model, the category of the 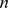
th patient is denoted by 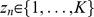 for 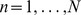. For that patient, 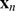 represents 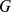-dimensional gene expression vector where the expression level of the 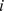
th gene is denoted by 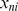 for 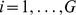. Similarly, 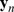 and 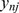 represent 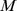-dimensional miRNA expression vector and the expression level of the 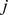 th miRNA for 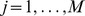 for the same patient, respectively. We have two different standard deviation parameters 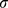 for genes (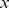) and miRNAs (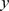) where each parameter is shared among the same type of transcripts. The mixture distribution for the 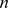
th patient can be written as
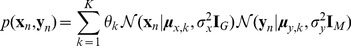
(4) where 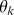 is the mixing coefficient such that 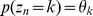 and the multivariate normal distribution parameters, 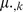 and 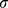, are the mean expression level vector of the 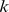
th subgroup and shared standard deviation, respectively.

The parameters can be learned from training data using the EM algorithm. The algorithm starts by initializing the model parameters to arbitrary values, and then the following steps are repeated until they reach a certain number of iterations or the parameters converge to the maximum likelihood values.

- ***E*** **-Step**: compute likelihood of 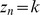 for 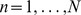 and 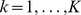.
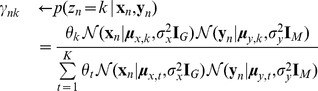
(5)
- ***M*** **-Step**: update 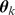, 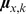, 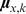, 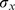 and 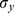 for 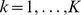.
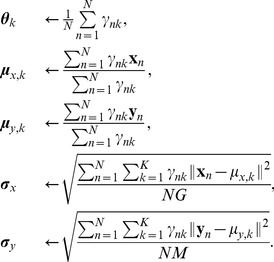
(6)

Finally, the class predictions can be made using the final likelihood values. The 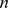
th patient was assigned to the subgroup 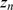 such that 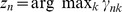, for 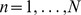. The algorithm can be run several times with different initializations to find the best parameters that maximize the likelihood of the model. The number of clusters, 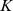, can be determined using Bayesian Information Criterion (BIC) [25]. The model that maximizes BIC is chosen among the models trained with different numbers of clusters.

Finally, we evaluated the cancer subgroups identified by our algorithm in computational and biological aspects. We carried out an assessment of the robustness of the cancer subgroups using bootstrap prediction analysis. We followed the procedure described in [26]. A subset of the patients was randomly resampled several times and the clustering algorithm was performed on each subset. Let 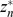 denote the class assignment of the 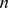
th patient by the mixture model trained on the entire dataset. Suppose that the same patient was selected for the 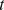
th subset and 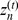 denote the class assignment of that patient by the model trained on the 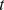
th subset. Then, the stability of class prediction for the 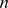
th patient was calculated as
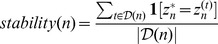
(7) where 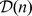 represents the set of all datasets in which the 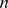
th patient is selected, and 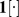 is an indicator function that returns 1 if the condition in square brackets is satisfied, and 0 otherwise. A stable clustering algorithm has stability scores close to 1 for every patient. For each patient, the proportion of class predictions was calculated and compared with the original predictions made from the entire dataset thus assessing the effectiveness of the method in both aspects. The biological evaluations comprised survival analyses and pathway analysis. We employed the Kaplan-Meier survival analysis for survival characteristics of patient clusters using the Prism software GraphPad (version 6, http://www.graphpad.com) and the Cox-Mantel log-rank test was used to determine the statistical significance [27]. Sensitivity analysis on iSubgraph parameters and comparison with an integrative approach were made too. We investigated if the genes and miRNAs in frequent subgraphs have been reported in previous studies as playing a role in cancer development using Ingenuity Pathway Analysis (IPA, Ingenuity Systems, http://www.ingenuity.com) that calculates the statistical significance of a particular gene and/or miRNA set in being associated with known pathways.

## Results

### Study Design and miRNA-mRNA Network Construction

**Figure 4:**
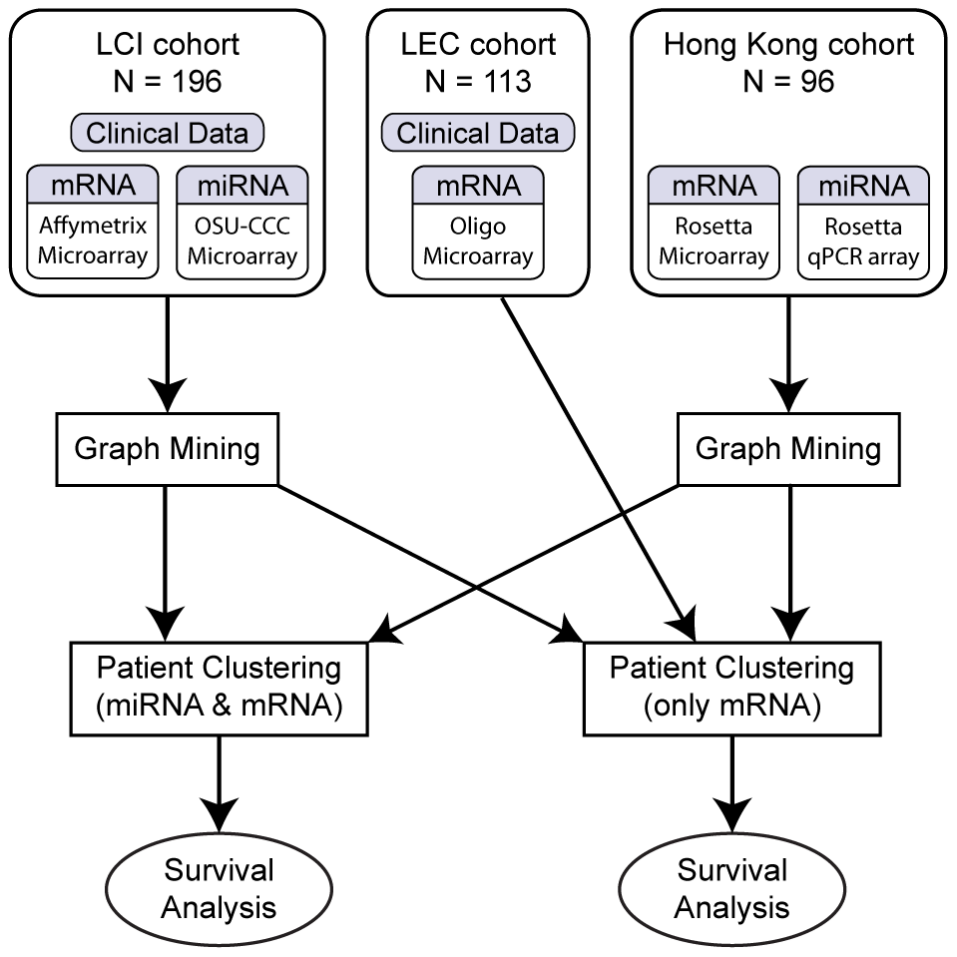
Schematic overview of the study design. Significant genes and miRNAs for HCC were identified separately for the LCI and Hong Kong cohorts using the graph mining approach of iSubgraph. The included genes and miRNAs were used as features in the subsequent clustering step of iSubgraph for the LCI and LEC cohorts. Finally, computational and biological analyses were carried out on the selected genes/miRNAs and cancer subgroups.

We applied the iSubgraph algorithm on two independent HCC cohorts to identify functionally distinct patient subgroups. The LCI and Hong Kong datasets were used to discover miRNA-mRNA co-modules and tumor subclassification. In addition to these two datasets, we used the LEC dataset, which includes only gene expression data, for validation. Frequent subgraphs extracted from the LCI and Hong Kong datasets were used separately for clustering the LCI and LEC cohorts. By using independent cohorts for clustering, we were able to validate the effectiveness of our gene and miRNA selection method by graph mining. Lastly, we performed survival analysis on the identified HCC subgroups. Clustering and survival analysis were not performed on the Hong Kong dataset due to lack of clinical data (Figure 4).

The first phase of the experiments was to find HCC associated miRNA-gene networks from the LCI and Hong Kong datasets using graph mining. We started this step by constructing the template bipartite graphs. We obtained miRNA targets from the TargetScan database (release 6.2, http://www.targetscan.org/vert_61) [19]. Next, we removed miRNA-mRNA pairs, which were not present in our datasets. There were 97,872 and 109,863 remaining miRNA-mRNA pairs in the LCI and Hong Kong datasets, respectively. After the correlation analysis with false discovery correction (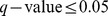), the resulting bipartite graph of the LCI dataset had 13,101 gene and 206 miRNA nodes with 20,323 edges. Similarly, the graph for the Hong Kong dataset had 18,503 gene and 202 miRNA nodes with 45,419 edges. The graphs had a substantial number of single isolated nodes with no edges. Therefore, these nodes were taken away from the graphs before graph mining to reduce computational time since they would not be found in frequent subgraphs due to the minimum number of gene nodes constraint.

### Patient Graph Construction

The sources of edges in patient graphs were the template bipartite graphs; however, for node tags we needed to decide over- and under-expressed cases of each gene and miRNA. We aimed to give equal chance to each gene and miRNA to be found in patient graphs. Therefore, each gene and miRNA had distinct cutoff values. We determined if a gene or miRNA is over- or under-expressed in a tumor sample by comparing its expression level with the average expression level of all samples (tumor and adjacent nontumor tissues) independently of all other transcripts. Let 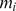 be the average expression level for the 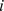
th gene, and 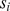 be the sample standard deviation of the same gene computed from all samples; then a patient graph contained a graph node for the 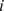
th gene, attached with an UP tag if the 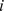
th gene was expressed greater than 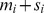 in that patient. Similarly, a patient graph had a node for that gene with a DOWN tag if its expression level was less than 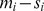. Otherwise, the node was removed from the patient graph. In other words, we used 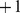 and 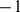 as 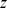-score thresholds for over- and under-expressed cases, respectively. We avoided making judgments on missing values using nearest neighbors, so we assumed they were neither up- nor down-regulated. Patient graph sizes appeared to be slightly smaller in the LCI cohort compared to the Hong Kong cohort (Figure S1). Thus, this method was able to incorporate up- and down-regulated genes without requiring a complete data set.

### Identification of miRNA-Driven Regulatory Networks

**Figure 5:**
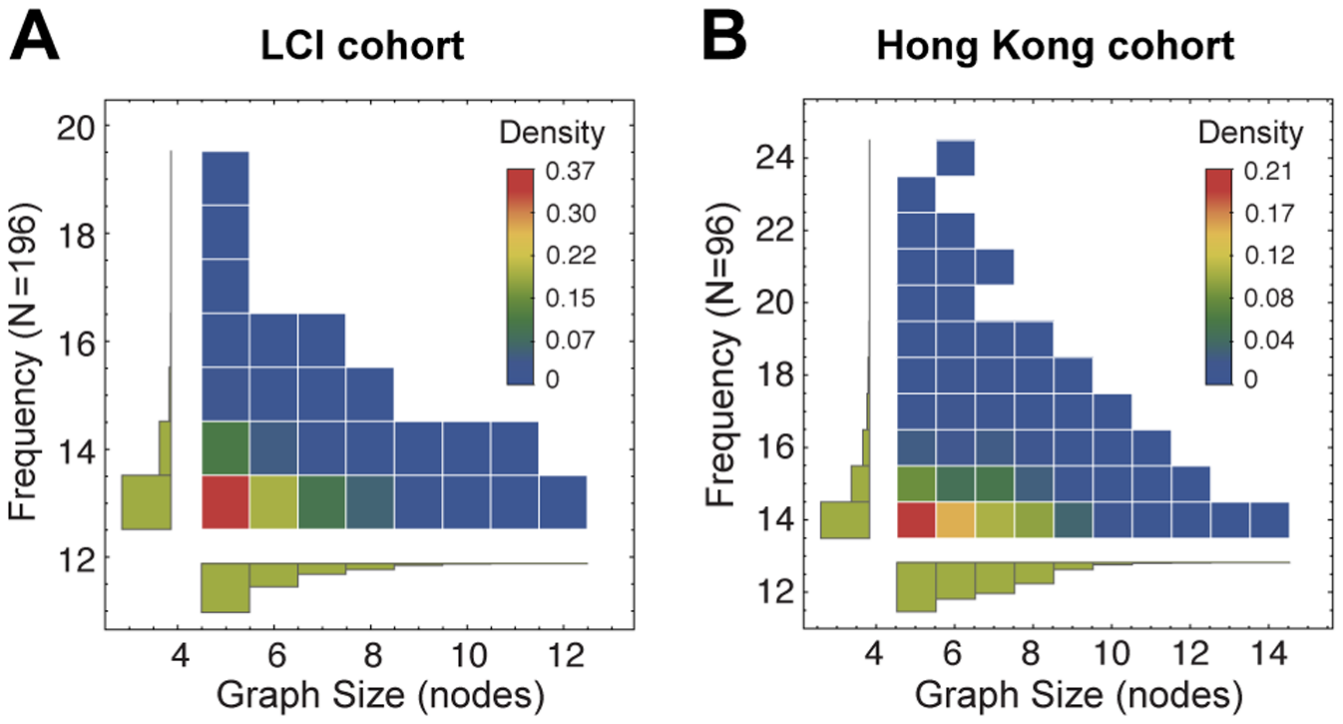
Frequency and graph size density of frequent subgraphs. (A) Density histograms of the frequent subgraphs from the LCI cohort (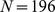) and (B) Hong Kong cohort (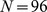) with respect to frequency and graph size. The frequency of a subgraph equals to the number of patients who have that subgraph in their patient graph.

We employed the MoSS software implemented by Borgelt for mining frequent subgraphs [28]. It was originally developed for finding frequent molecular substructures but it can be adapted for other data types as well (for details see Materials and Methods). For a given graph set, the graph mining software only requires graph mining parameters to find all closed frequent subgraphs. The number of frequent subgraphs returned by the graph mining algorithm depended on support thresholds and microarray platforms. They also affected the computational time required for graph mining and the following steps. Therefore, we determined the support thresholds experimentally that yielded around 5000 most frequent closed subgraphs. The number of genes in the Hong Kong dataset was greater than that of the LCI dataset. Correlation coefficients between miRNAs and their target genes were usually greater in the Hong Kong dataset too. Consequently, we had to use a larger support threshold for the Hong Kong cohort to obtain similar numbers of frequent subgraphs. The support thresholds were 13 (6.6%) and 14 patients (14.6%) for the LCI and Hong Kong cohorts, respectively. For both datasets, we removed frequent subgraphs that had less than 4 gene nodes (Figure 5). The numbers of remaining closed frequent subgraphs were 6,280 and 4,418 in the LCI and Hong Kong datasets, respectively. All frequent subgraphs were found statistically significant by multiple hypotheses testing. Most of the miRNAs in the subgraphs were differentially expressed in tumor samples. Thirty-nine out of 49 miRNAs for the LCI cohort and 40 out of 46 miRNAs for the Hong Kong cohort were differentially expressed (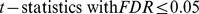). Each step of frequent subgraph discovery functioned as a filter for genes and miRNAs. Table 2 provides the residual numbers of genes and miRNAs in each datasets after each step. Hence, we were able to narrow down the sets of genes and miRNAs to a short list of HCC-associated transcripts for the mixture model.

**Table 2:** Number of genes and miRNAs after each graph mining step.

	LCI Cohort		Hong Kong Cohort	
Step	#genes	#miRNAs	#genes	#miRNAs
Initial data	13,101	206	18,503	202
In TargetScan Predictions	7,303	206	9,529	202
After miRNA-mRNA correlation analysis	4,401	171	7,572	200
Based on support threshold	4,374	171	7,399	194
In frequent subgraphs	384	49	418	46

**Figure 6:**
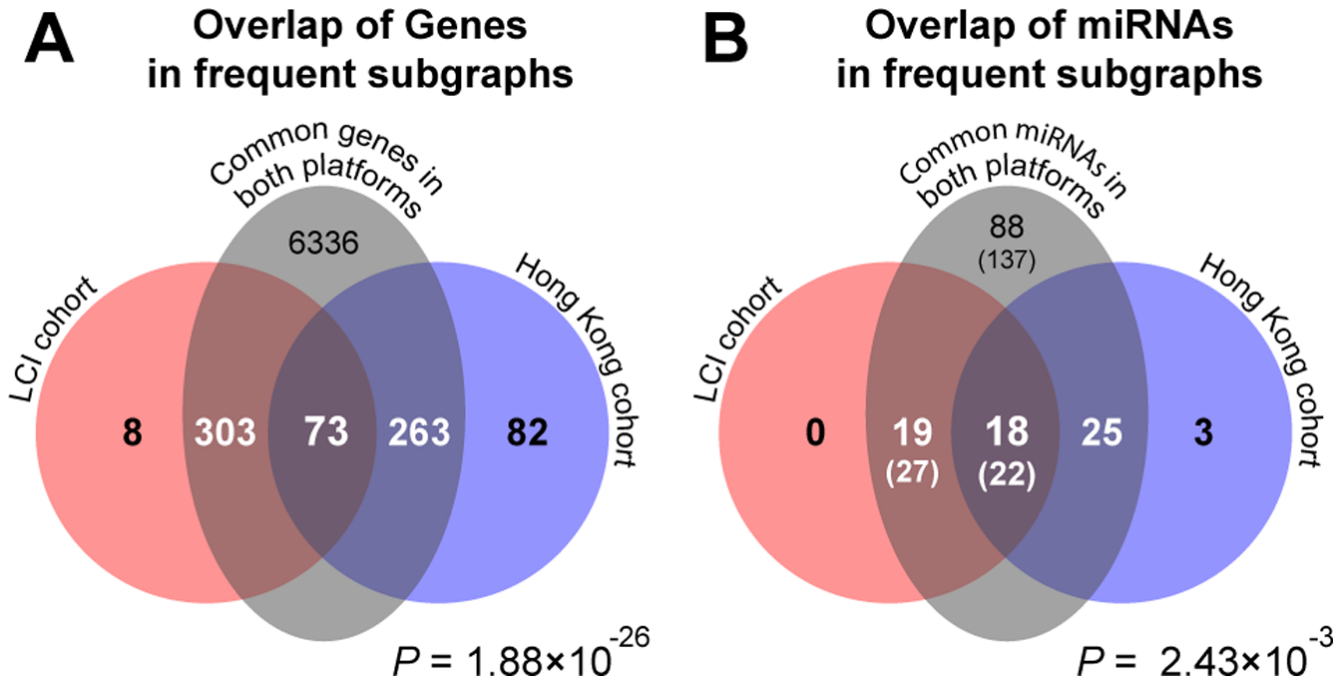
Overlapping genes and miRNAs in subgraphs of the LCI and Hong Kong cohorts. (A) Euler Diagrams showing the overlap between the genes and (B) miRNAs occurring in frequent subgraphs from the LCI and Hong Kong datasets. A single miRNA probe in the Rosetta platform (Hong Kong cohort) may correspond multiple probes in the OSU-CCC platform (LCI cohort). The numbers in parentheses show the probe counts in the LCI data. P-values were computed from hypergeometric tests. The number of common genes and miRNAs indicate solely those transcripts that were present in the TargetScan database.

**Figure 7:**
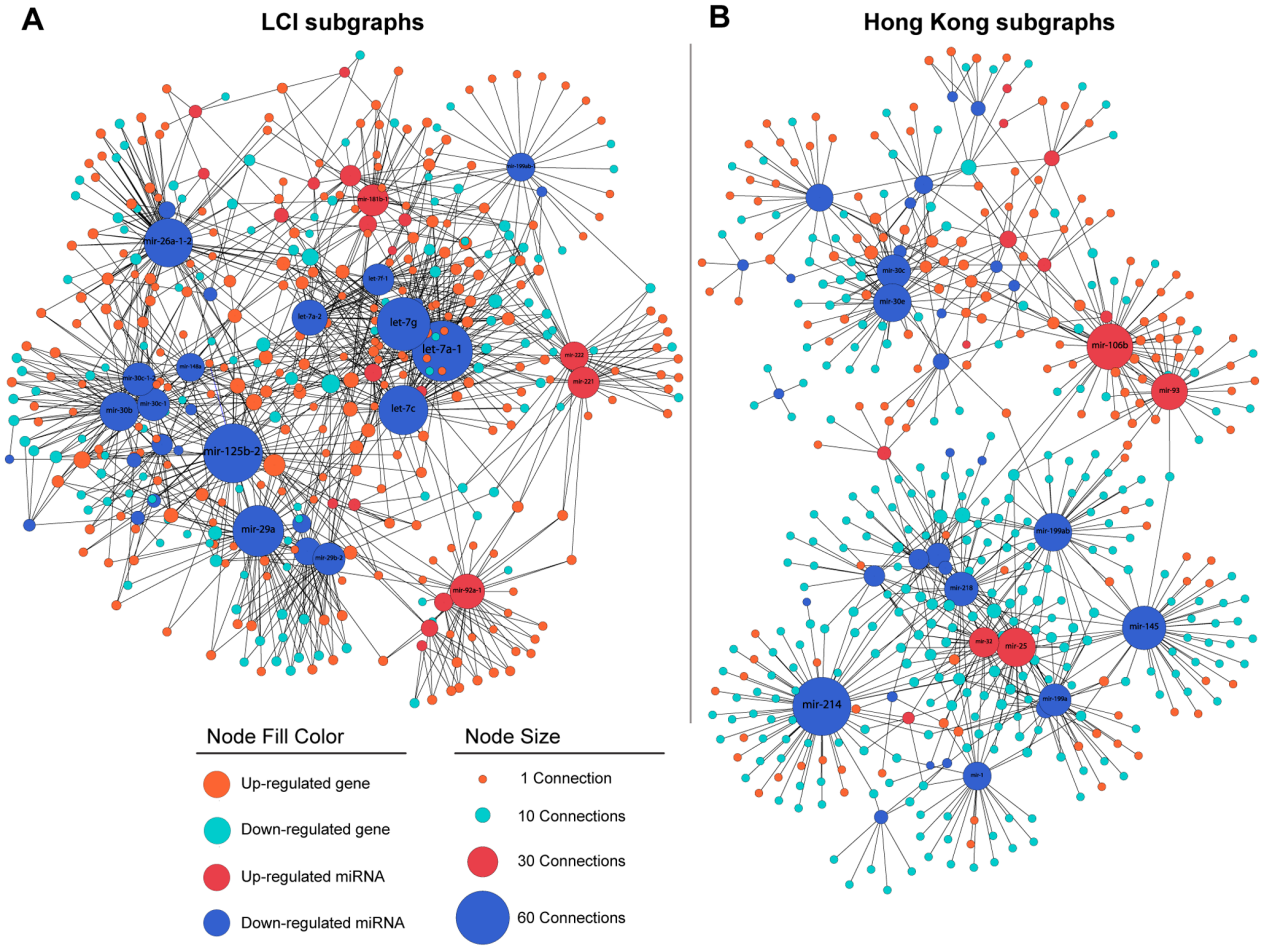
Regulatory Networks associated with HCC. (A) MiRNA-mRNA networks from the LCI and (B) Hong Kong datasets. The networks were constructed by merging vertices and edges of all frequent subgraphs from those datasets. The node color represents the transcript and regulation type. The node size indicates the number of connections. Unlike the current layout of the nodes for visualization purposes, both networks are originally bipartite graphs.

Although the LCI and Hong Kong cohorts have been collected in different hospitals, during different time frames and also the microarray data has been obtained on different platforms, the frequent subgraphs from the LCI and Hong Kong cohorts had a considerable number of common nodes. This relationship is shown by Euler Diagrams (Figure 6). Figure 7 displays the combined miRNA-mRNA regulatory networks of all frequent subgraphs for each cohort. In the LCI cohort the let-7 family, miR-125b, miR-26, miR-29 and miR-30 are down regulated and appear to be key regulatory elements. On the other hand, in the Hong Kong cohort miR-214, miR-145, miR-199 and miR-30 which are down and miR106b which is up regulated have the most connections. Therefore, it appears that of the five miRNAs with the most connections both cohorts share only miR-30. Thus, the main regulatory miRNAs in the LCI and Hong Kong cohort differ significantly.

### Identification and Stability of Patient Subgroup Prediction

**Figure 8:**
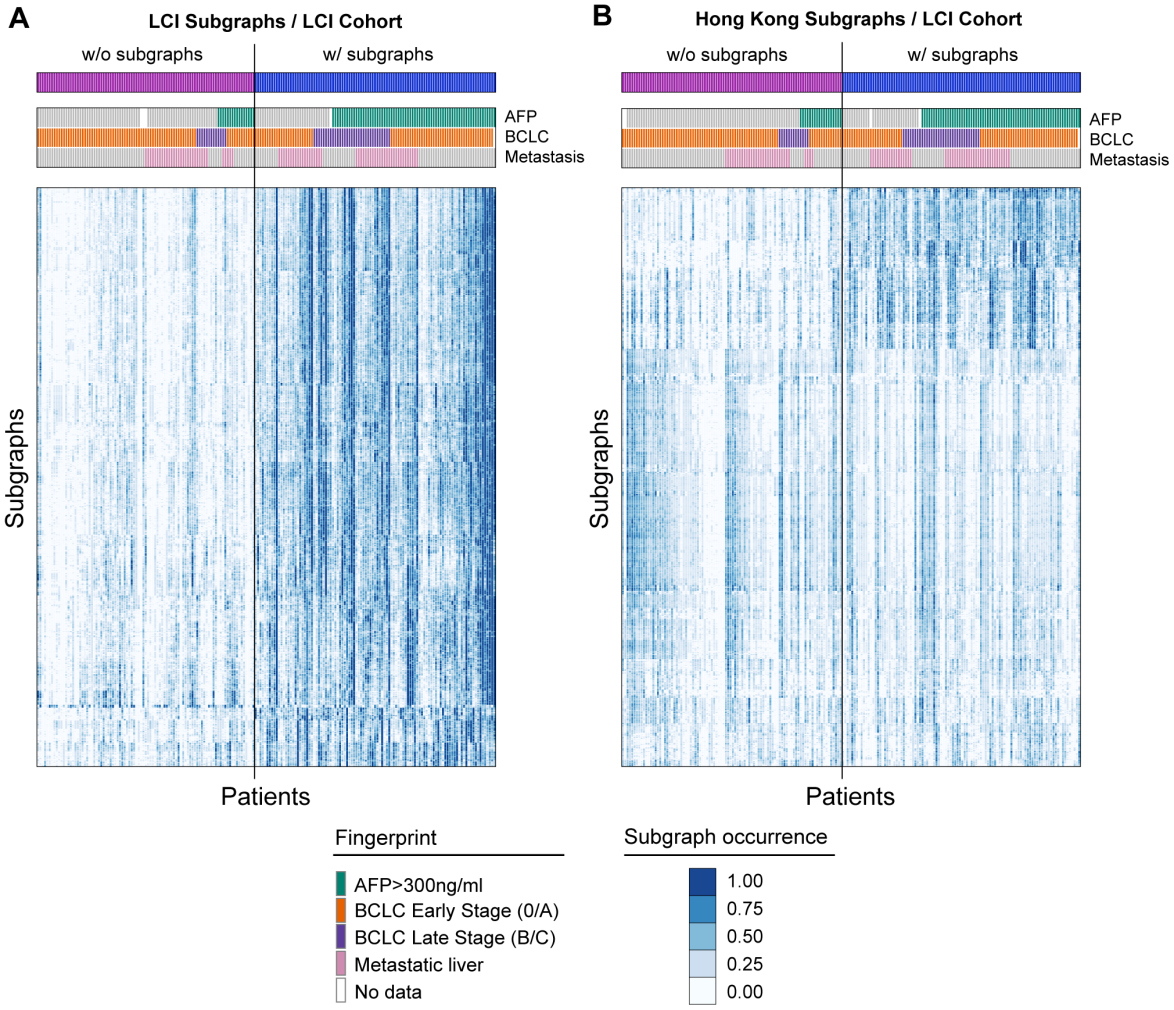
Clinical properties and heatmap of subgraph occurrence in the LCI cohort. (A) Occurrence of all subgraphs identified in the LCI and (B) Hong Kong cohorts. The order of patients in subgraph occurrence heatmap (bottom) is arranged according to class assignments (top) and clinical properties (middle). Subgraph occurrence in a patient is defined as the ratio of common nodes between the subgraph and the patient graph to all nodes of that subgraph. Abbreviations: AFP, Alpha-fetoprotein; BCLC, Barcelona clinic liver cancer.

The second phase of the experiments was to cluster HCC patients by mixture models using the subgraphs extracted from the patient graphs. Graphs provide powerful structural models that not only represent the values of expression profiles, but can also be used to explicitly model relations that exist between different transcripts. However, their use for clustering has been limited due to difficulties and inefficiencies in structure-based comparisons of these graphs. The alternative approach is embedding graphs into vector space and using an algorithmic tool for pattern analysis from the rich repository of statistical machine learning. For example, the graph edit distance with dissimilarity representation or Lipschitz embedding work well for matching relatively small graphs [29], [30]; however, it can become quite restrictive for graphs with a large number of nodes and edges such as patient graphs. Another approach is to use the occurrence data of frequent subgraphs in patient graphs as features to a clustering method. However, this may lead to unstable subgroup predictions because of the redundancy between frequent subgraphs, the strict rules of subgraph isomorphism and the dependency of labeling function on thresholds. The redundancy in occurrence data of subgraphs can be seen in Figure 8. There are 6,280 closed frequent subgraphs mined from the LCI dataset and all of those subgraphs have a total of 433 distinct vertices (384 genes and 49 miRNAs). This shows that a gene or miRNA node appears on average on 14.5 different subgraphs. As a result, merging all subgraphs into a single co-module and using the expression values of the genes and miRNAs in that co-module for patient stratification aided eliminating the redundancy among subgraphs and reducing the dimensionality of features. Besides, this co-module exhibited a more generalized HCC-related regulatory network.

In total, four distinct mixture models were trained to cluster the LCI and LEC cohorts using the frequent subgraphs from the LCI and Hong Kong datasets, respectively. The two models for clustering the LEC cohort had only gene variables. The Bayesian Information Criterion (BIC, see Materials and Methods for details) was maximized, for all four cases, when the patients were clustered into two groups. In Eq. (1), subgraph occurrence, 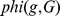, is defined as a binary function. For the analysis of patient characteristics in terms of expression levels, we defined a modified version of this function that assigns a score between 0 and 1 for each subgraph-patient pair, indicating the occurrence of a particular subgraph in a patient graph. Let 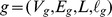 denote a subgraph and 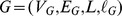 denote a patient graph, then the occurrence score of 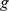 in 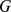 was computed as 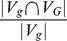, i.e. the ratio of common nodes between 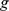 and 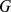 to all nodes of 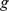. We observed that the patient graphs of one patient group in the LCI cohort mostly contained the frequent subgraphs compared to the other group. Therefore, we named the patient clusters as *subgroup w/subgraphs* and *subgroup w/o subgraphs* (Figure 8A). Interestingly, although the subgraphs of the Hong Kong cohort did not contain the same miRNA-mRNA networks, clustering of the LCI cohort using the Hong Kong subgraphs led to very similar clustering results (Figure 8B). Significant enrichment was determined for AFP (Alpha-fetoprotein) and BCLC (Barcelona clinic liver cancer) stage using Fisher's exact test right-tailed (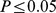) for both cases. However, metastasis failed to reject the null hypothesis in the same analysis (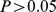). The subgroup w/subgraphs has more patients with metastasis, high AFP level and BCLC late stages. Therefore, the subgraphs identified in the graph mining step are associated with tumor progression.

We evaluated the robustness of clustering by mixture models. The stability of patient class assignments was measured on random datasets generated by resampling patients from the entire cohort. We followed the procedure described in [26]. For all mixture models, we generated 100 random datasets, each of which comprised 70% of the cohort, and we ran the mixture model based clustering scheme on each random dataset. The proportions of class predictions in random datasets were calculated for each patient and compared with the class assignments from the entire datasets (Figure S2). Most of the patients were repeatedly assigned to the same subgroups in all trials, i.e. the stability scores equal to 1 for most patients (Eq. (7), see Materials and Methods for details). In addition to the bootstrap stability analysis on random datasets, we compared the class assignments of an entire cohort by mixture models trained on different subgraphs. There were only 11 patients (5.61%) of the LCI cohort assigned to different subgroups by the models trained from the LCI subgraphs and the Hong Kong subgraphs. The similarity of clustering was measured by the Rand index as 0.894 and the adjusted Rand index was 0.787 [31]. For the LEC cohort, there were 7 patients (6.19%) stratified differently using those subgraphs. The Rand index was 0.883 for the LEC clusterings and the adjusted Rand index was 0.766. Therefore, clustering by the mixture model using the subgraphs from each dataset was very stable in both cohorts and resulted in high confidence of subgroups.

### Validation of Patient Subgroups in Two Independent Cohorts

**Figure 9:**
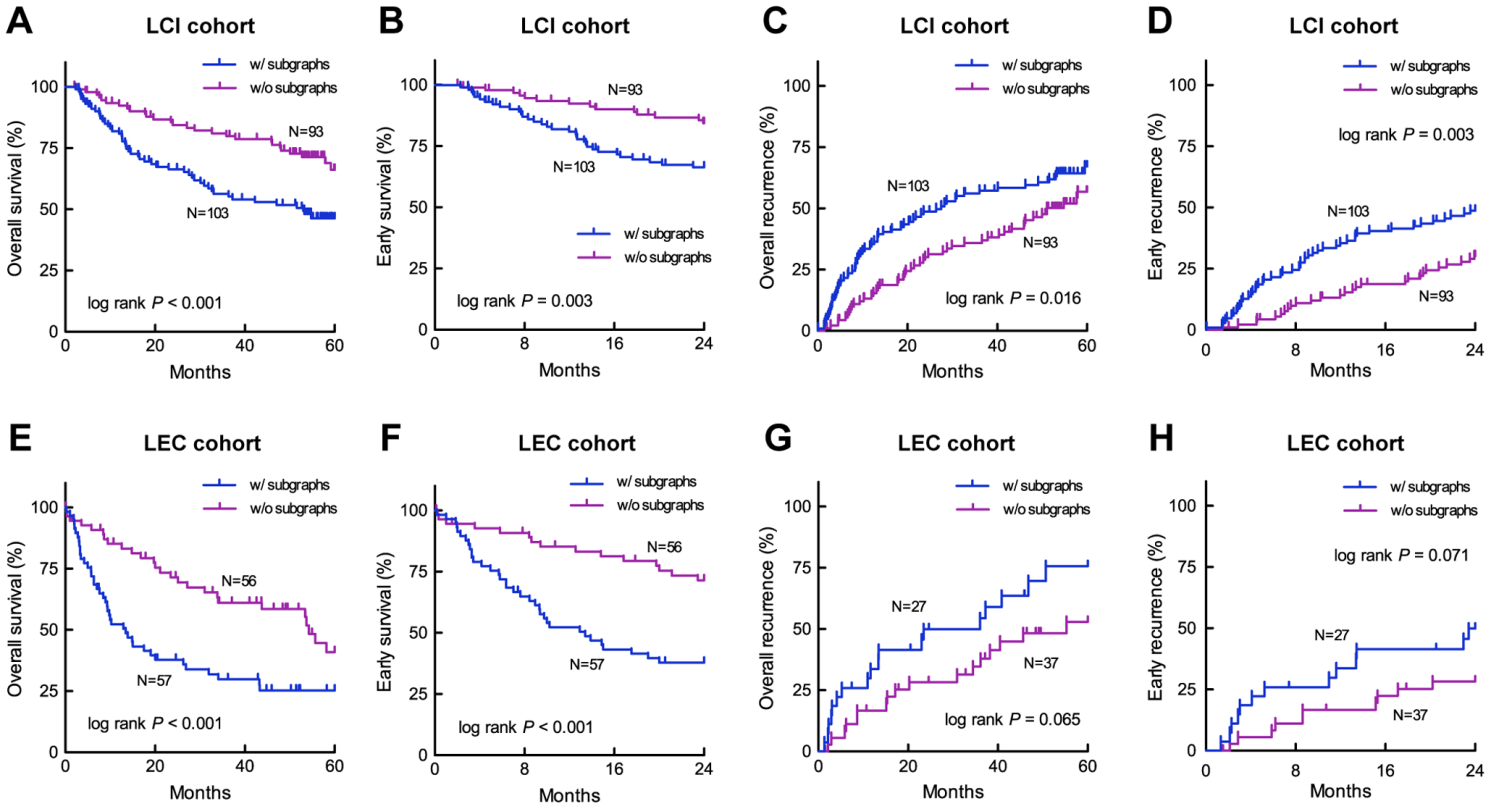
Combined Kaplan-Meier curves of patients subgrouped using the LCI subgraphs. The first row shows the survival and recurrence characteristics of the LCI cohort (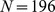) and the second row shows those of the LEC cohort (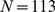). The recurrence information was not available for some patients of the LEC cohort. From left to right, the columns indicate the overall survival (survival rates in the first 5 years), early survival (survival rates in the first 2 years), overall recurrence (disease-free survival rates in the first 5 years), and early recurrence (disease-free survival rates in the first 2 years) curves. 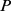-values were calculated by the Log-rank (Mantel-Cox) test.

We investigated the cancer subgroups identified by the iSubgraph algorithm in terms of survival and recurrence characteristics. These analyses demonstrated that the here developed method is able to separate patient subgroups into good and poor outcome groups in the LCI and LEC cohorts where the subgroups were determined by the LCI subgraphs (Figure 9). Thus, the survival analysis showed that our method was able to identify patient subgroups with significantly different overall survival and recurrence in the LCI cohort which could be validated in the independent LEC cohort. Likewise, similar results were obtained applying the Hong Kong subgraphs to the LCI and LEC cohorts for independent validation (Figure S3).

The next biological evaluation was pathway analysis to identify pathways enriched in the frequent subgraphs. In Ingenuity Pathway Analysis (IPA), the default settings were used where both direct and indirect relationships were considered for 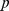-value calculations in networks and upstream regulator analysis. All genes and miRNAs in frequent subgraphs were used in the analyses separately for each cohort. The top five upstream regulators for each gene-miRNA set in the LCI and Hong Kong cohorts show very little overlap (Table 3). It appears that the only upstream regulator which is common to both cohorts is TP53. Similarly, there are two common pathways in the top ten signaling pathways of both cohorts. (Table 4). This confirms that the identified genes and miRNAs of the two cohorts share only minor similarities. Thus, although in both cohorts the identified patient subgroups differ significantly in survival, iSubgraph identifies distinct pathways that are independently survival associated.

**Table 3:** The most significant upstream regulators and corresponding 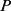-values calculated by Ingenuity Pathway Analysis.

LCI Cohort		Hong Kong Cohort	
Regulator	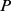 -value	Regulator	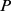 -value
Myc	2.52E-12	TGFB1	3.86E-18
E2F1	7.33E-10	TNF	3.47E-12
Let-7	7.75E-09	EGFR	1.79E-10
*TP53*	3.71E-08	*TP53*	1.01E-09
FGF21	5.04E-08	Prostaglandin E2	1.10E-09

**Table 4:** The most significant canonical pathways and corresponding 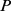-values calculated by Ingenuity Pathway Analysis.

LCI Cohort		Hong Kong Cohort	
Canonical pathway	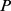 -value	Canonical pathway	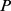 -value
Estrogen Receptor Signaling	3.80E-04	Axonal Guidance Signaling	1.32E-07
*ERK/MAPK Signaling*	7.08E-04	Hepatic Fibrosis/Hepatic Stellate Cell Activation	5.50E-06
4-aminobutyrate Degradation I	1.32E-03	Regulation of the Epithelial-Mesenchymal Transition Pathway	6.61E-06
Glucocorticoid Receptor Signaling	1.45E-03	Cardiac Hypertrophy Signaling	6.76E-06
*Estrogen-mediated S-phase Entry*	1.51E-03	*Estrogen-mediated S-phase Entry*	1.58E-05
Role of BRCA1 in DNA Damage Response	1.66E-03	PKC 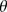 Signaling in T Lymphocytes	2.00E-05
Estrogen-Dependent Breast Cancer Signaling	2.14E-03	Role of NFAT in Cardiac Hypertrophy	2.95E-05
TR/RXR Activation	2.19E-03	*ERK/MAPK Signaling*	4.27E-05
Biotin-carboxyl Carrier Protein Assembly	2.63E-03	Breast Cancer Regulation by Stathmin1	5.75E-05
Protein Kinase A Signaling	3.24E-03	Molecular Mechanisms of Cancer	1.95E-04

### Sensitivity Analysis on Parameters and Comparison with Other Methods

Our final experiments were assessing the sensitivity of clustering performance on parameter selection and comparing iSubgraph with a recent integrative approach. The parameters of iSubgraph can be grouped into four categories. These are expression level cutoff values for tag assignments (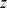-score thresholds), minimum support threshold for graph mining, minimum number of gene nodes in frequent subgraphs, and the number of subgroups. A set of parameters can be denoted by a 4-tuple in the same order, e.g. 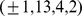 for the case in Figure 9A. The parameter for the number of subgroups can be determined by Bayesian information criteria or a pre-defined number can be used. We analyzed the sensitivity of iSubgraph to each parameter using one-at-a-time perturbation except z-score thresholds, which required additional support threshold adjustments to get sufficient number of frequent subgraphs. We considered the survival curves in Figure 9A as the baseline in sensitivity analysis and compared them with the survival curves of different parameter settings (Figure S4). Although small changes in support threshold affected the number of subgraphs and computational time exponentially (Figure 5), the alterations were comparatively minor in the set of genes and miRNAs that were present in the frequent subgraphs (Table S1). The survival curves remained significantly distinct on small parameter perturbations because the set of genes and miRNAs identified by iSubgraph was highly correlated and removing some of them did not change the class assignments by the mixture model dramatically. A value around 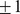 for the 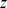-score threshold might give sufficient number of subgraphs including significant genes and miRNAs for many cases. Therefore, we suggest parameter settings on which the graph mining algorithm terminates in short time, and the frequent subgraphs include a few hundred or less genes and miRNAs. It would be safe to start with a large support threshold that will eventually decrease to a reasonable value. Hence, the survival curves in Figure S4 showed that different parameter settings had little effect on the performance of the iSubgraph algorithm.

Before comparing iSubgraph with an integrative approach, we assessed if the integration of gene and miRNA expression values really lead to improvements. The LCI cohort was divided into two groups using the mixture models trained solely on the expression values of all genes. The same procedure was repeated for the expression values of all miRNAs. Then, the mixture model was also trained on the occurrence information of subgraphs found by the graph mining algorithm. Finally, the survival curves of all these classifications were compared with that of iSubgraph (Figure S5). Using only miRNA expression failed to give significantly distinct survival curves; however, the mixture model managed to divide patients into two groups with different survival characteristics using only gene expression data. The separation in survival curves between two classes was much improved by the iSubgraph algorithm. The usage of subgraph occurrence in tumor subclassification is also an integrative approach; however, it suffered from problems explained before in the Results section. Thus, taking the iSubgraph approach, which is using the selected features of integrated expression data in the training of the mixture model, helps to overcome the limitations of subgraph occurrence data and to improve the performance of tumor subclassification.

**Figure 10:**
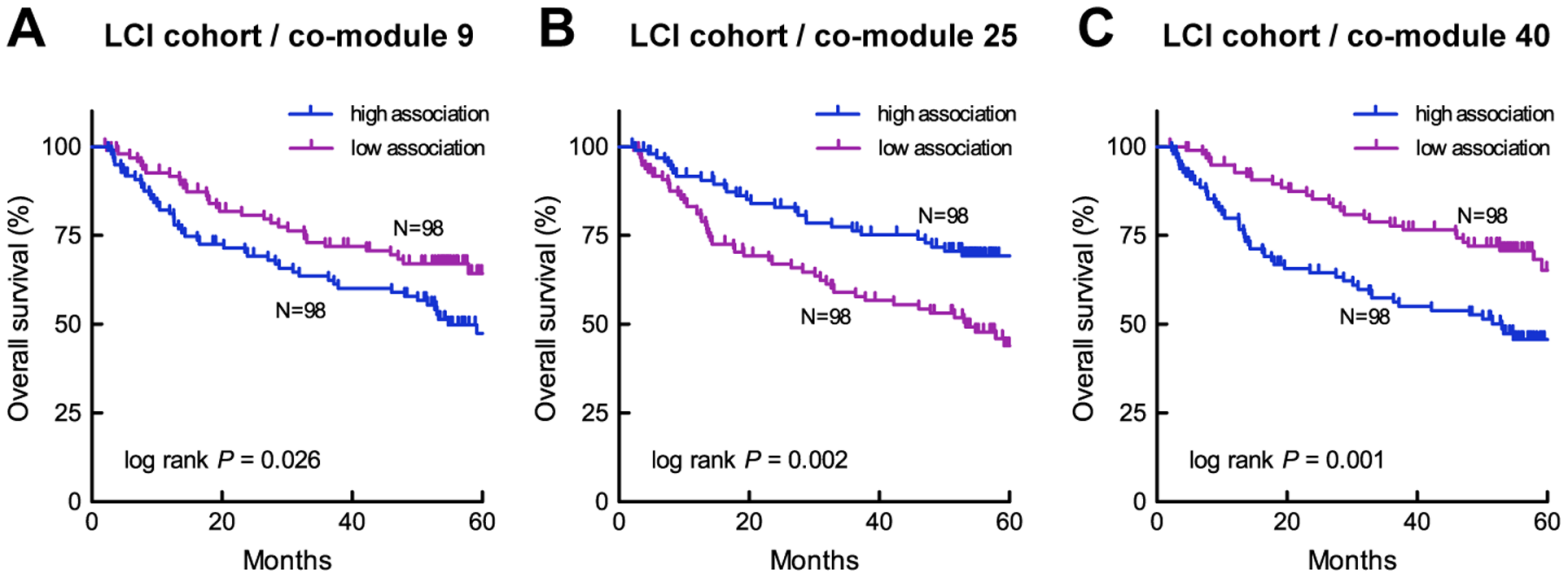
Kaplan-Meier curves of the LCI cohort subgrouped by SNMNMF modules. Survival curves in the first 5 years are shown for three different subgrouping of the LCI cohort (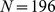). The patients are divided into two equal size groups with respect to their signals in the co-module basis vectors. Shown are the three co-modules which exhibit the most significant differences in their clinical parameters among all co-modules. 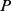-values were calculated by the Log-rank (Mantel-Cox) test.

We compared the iSubgraph algorithm with a recent integrative approach by Zhang et al. [22]. This approach proposes patient classification based on their association with miRNA-gene regulatory co-modules, which are identified using the sparse network-regularized multiple non-negative matrix factorization (SNMNMF). The miRNA and gene expression matrices are factorized into a common basis 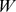 and two coefficient matrices 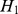 and 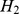. This process involves an optimization problem with network (gene-gene and miRNA-gene interactions) and sparsity constraints. The co-modules are identified based on association values in the coefficient matrices and patients are grouped based on association values in the basis matrix. The performances of the iSubgraph and SNMNMF methods in tumor subclassification were compared on the LCI dataset. Before applying the SNMNMF method on the LCI dataset, missing values in the miRNA profiles were estimated using 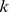-NN imputation [32]. We used the same parameter values as in [22]. Among the 50 co-modules identified by the algorithm, 4 were empty and therefore deleted, 39 contained only genes, and the remaining 7 had a single miRNA. These results may stem from relatively low correlation values between genes and miRNAs in the LCI cohort. In [22], patients are divided into three groups independently by each co-module. Each subgrouping is tested for distinct survival characteristics using calculating Kaplan€Meier curves. However, in this study we divided patients into two equal groups based on the median values of basis vectors in 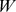 in order to make a direct comparison between the subclassification of the LCI cohort by iSubgraph. Eight co-modules exhibited significant differences in their survival curves (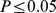). The Kaplan-Meier curves of the most significant three classifications can be seen in Figure 10. The major drawback to comparing the overall performances of both algorithms is that there is no single stable clustering result provided by the SNMNMF method. Among 50 co-modules, only two co-modules (25 and 40) provided significantly distinct survival curves close to those of iSubgraph. We additionally analyzed the genes and miRNAs in the co-modules of SNMNMF. There were 7 miRNAs and 390 genes in all co-modules; however, the overlap between the transcripts identified by SNMNMF and iSubgraph was very limited: 21 genes and no miRNA. Moreover, no common top upstream regulator or canonical pathway was found in IPA. The main reason for lack of overlap was that the genes identified by SNMNMF had low correlation with miRNAs and most co-modules of SNMNMF included only genes with high correlation. On the other hand, iSubgraph seeks only correlated miRNA-gene pairs in frequent subgraph mining. Therefore, iSubgraph enriches genes and miRNAs that are most likely to be functionally linked due to target gene prediction by TargetScan and actual correlation of expression.

## Discussion

The amount of microarray data for both gene and miRNA expression is constantly increasing. Despite the significant role of miRNAs in tumor development, the knowledge about the regulation between miRNAs and genes has remained limited. The availability of large amounts of genomics data such as the TCGA project has enabled the study of integrated analysis for certain cancers [33]. However, the efforts to integrate gene and miRNA data have been limited for tumor subclassification.

In recent years, a few computational methods have been proposed to identify miRNA-mRNA regulatory networks. Yoon and De Micheli introduced the first method to predict miRNA regulatory modules (MRMs) [34]. They defined data mining tasks for miRNA-mRNA binding sites at sequence level and represented the relations between miRNAs and target genes by a weighted bipartite graph. However, using only seed-based predictions for miRNA targets has been considered inadequate to determine combinatorial interactions between miRNAs and genes in a specific biological process. More recent methods have taken miRNA and gene expression profiles into account to improve target predictions. Several strategies have been adopted for discovery of miRNA-mRNA interactions from microarray data. The fusion models proposed for miRNA and gene expression data embrace a probabilistic graphical model [35], a co-evolutionary learning algorithm [36], a rule-based learning approach [37], a Bayesian network structure learning with splitting-averaging strategy [38], layered hyper-networks [39] and a graph theoretical approach by generating all maximal bi-cliques as candidate regulatory modules [40]. However, these methods construct a single model that considers all patients coming from a single group without taking the heterogeneity among patients into account, i.e. the possibility that multiple patient subgroups exist.

In this study, we developed the iSubgraph algorithm to investigate miRNA-driven regulatory mechanisms and their association with cancer subgroups. In the first part, we analyzed the patterns of miRNA-mRNA regulations in two HCC datasets. The subgraph mining approach effectively identified common regulation patterns among liver cancer patients. The computational analysis of the clustering method by randomization reveals the high stability of the method for all cases (Figure S2). Moreover, clustering comparisons by Rand index showed large agreements between two clusterings from different subgraphs. This property ensures that applying this method on other HCC datasets with similar clinical properties can give similar tumor subclassification even if the number of common genes is relatively small (Figure 6). This stability was satisfied by the usage of shared variance parameters because they restricted the effects of genes and miRNAs with small variance, in other words noisy features, on clustering. Our experiments using different mixture models with multiple variance parameters (covariance matrix) performed less stable predictions because of the limited number of training samples compared to the number of parameters (data not shown). In addition, identifying highly correlated gene-miRNA networks by graph mining repeatedly provided significantly distinct survival curves in sensitivity analysis in spite of the changes in the set of selected genes and miRNAs (Figure S4). The Kaplan-Meier survival analyses in Figure 9 draw the conclusion that the subgroups identified by the algorithm have distinct survival characteristics. Moreover, the integrated analysis led substantial improvements in class predictions made from miRNA expression profiles. There were also slight improvements for gene expression profiles (Figure S5).

As the amount of available genomic data increases, integrated analysis of multiple whole genome types gains more interest. The iSubgraph algorithm can be easily extended to other omics types such as DNA methylation, histone modification and metabolomics. New omics data can be attached as a new layer of nodes to the previous bipartite graph. Then, the nodes are assigned with appropriate tags and they are connected by edges to the nodes of other omics types that they interact with. The graph mining algorithm can handle the discovery of regulatory networks including the additional data type. Similarly, adding new variables, depicted as shaded circles in the graphical model, to the mixture model and updating the probability equations accordingly are sufficient for clustering patients by multiple omics dimensional data (Figure 3).

The genes and miRNAs found in the subgraphs exhibit dissimilar patterns of occurrence in patients and different level of interactions between each other in the LCI dataset and Hong Kong dataset. As seen in Figures 7 and 8, the subgraphs of the LCI dataset were moderately close to each other and they were found in the same patients. On the other hand, the subgraphs of the Hong Kong dataset show two different groups of subgraphs; however, one group is more explicit in the subgraph occurrence matrix. The mixture model generally grouped the patients according to this property. The subgroup w/subgraphs included more patients with high AFP, metastasis or BCLC late stage (Figure 8). Thus, the iSubgraph algorithm was effective in identifying stable subgroups in two independent cohorts. In addition, further functional studies of the integrated miRNA-mRNA networks may improve our understanding of HCC development and progression and may lead to the development of targeted therapies specific to patient subgroups.

## Supporting Information

Procedure S1 **Correlated Target Prediction Method.**
(PDF)

Procedure S2 **Patient Graph Construction Method.**
(PDF)

Figure S1
**Histograms of patient graphs.** (A) Histograms of patient graphs from the LCI (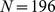) and (B) Hong Kong cohort (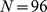) with respect to graph size. The red dashed curves, which are cut due to visualization purposes, show expected values.(TIF)

Figure S2
**Robustness of class predictions.** The proportion for each sample was calculated based on bootstrap prediction analysis (by resampling 70% of patients 100 times). The order of patients (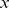-axis) is arranged according to proportions of class prediction in each panel. (A) Panels on the left show prediction analysis of the LCI cohort for the subgraphs from the LCI cohort and (C) Hong Kong cohort. (B) Panels on right show prediction analysis on the LEC cohort for the subgraphs from the LCI and (D) Hong Kong cohorts. Class assignments by the mixture models trained on entire dataset are shown below each plot.(TIF)

Figure S3
**Combined Kaplan-Meier curves of patients subgrouped using the Hong Kong subgraphs.** The first row shows the survival and recurrence characteristics of the LCI cohort (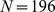) and the second row shows those of the LEC cohort (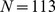). The recurrence information was not available for some patients of the LEC cohort. From left to right, the columns indicate the overall survival (survival rates in the first 5 years), early survival (survival rates in the first 2 years), overall recurrence (disease-free survival rates in the first 5 years), and early recurrence (disease-free survival rates in the first 2 years) curves. 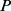-values were calculated by the Log-rank (Mantel-Cox) test.(TIF)

Figure S4
**Kaplan-Meier curves of the LCI cohort subgrouped with small parameter perturbations.** Survival curves in the first 5 years are shown for the LCI cohort (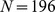) subgrouped by the mixture model. The parameter setting in Figure 9A is used as a reference, where 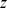-score cutoffs, minimum gene node count, support threshold and number of subgroups were set to 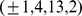, respectively. The experiments were repeated with perturbations in each panel, namely (A) decreasing the minimum gene node count to 3, (B) increasing the support threshold to 14, (C) decreasing the 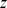-score thresholds to 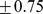 and increasing the support threshold to 21, (D) increasing the number of subgroups to 3, (E) increasing the minimum gene node count to 5, (F) increase the support threshold to 15, (G) increasing the 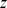-score thresholds to 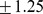 and decreasing the support threshold to 8, (H) increasing the number of subgroups to 4. 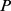-values were calculated by the Log-rank (Mantel-Cox) test.(TIF)

Figure S5
**Kaplan-Meier curves of the LCI cohort subgrouped using different data types.** Survival curves in the first 5 years are shown for the LCI cohort (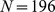) subgrouped by the mixture model trained on (A) expression data of genes only, (B) expression data of miRNAs only, (C) occurrence data of the LCI subgraphs, and (D) expression data of genes and miRNAs found in the LCI subgraphs. 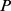-values were calculated by the Log-rank (Mantel-Cox) test.(TIF)

Table S1 **The number of closed frequent subgraphs and the number of genes and miRNAs in those subgraphs for different parameter settings in the LCI dataset.**
(PDF)
