# Supplementary material for: iSubgraph: Integrative Genomics for Subgroup Discovery in Hepatocellular Carcinoma Using Graph Mining and Mixture Models
Source: PLoS One. 2013 Nov 4;8(11):e78624. doi: 10.1371/journal.pone.0078624 (PMC3817163; doi:10.1371/journal.pone.0078624)
Supplement: Procedure S1 — Correlated Target Prediction Method. (PDF) [file pone.0078624.s001.pdf]

Procedure S1 Correlated Target Prediction Method

```
Input: Gene expression data, {x_ni},
       miRNA expression data, {y_nj},
       seed-based target predictions {c_ij}.
Output: Correlated target predictions {c*_ij}.
  ▷ Compute correlation matrix and P-values
  for each pair (i, j) do
      u ← (x_1i, x_2i, ..., x_Ni)^T
      v ← (y_1j, y_2j, ..., y_Nj)^T
      R_ij ← Correlation(u, v)
      P_ij ← 0
      for t ← 1 to 1000 do
          u' ← Permute(u)
          v' ← Permute(v)
          R'_ij ← Correlation(u', v')
          if R'_ij ≤ R_ij < 0 or R'_ij ≥ R_ij > 0 then
              P_ij ← P_ij + 1/1000
          end if
      end for
  end for
  ▷ Assign correlated targets
  for j ← 1 to M do
      S ← {P_ij | c_ij = 1 and i = 1, ..., G}
      cutoff ← FDR(S, 0.05)
      for i ← 1 to G do
          if c_ij = 1 and P_ij ≤ cutoff then
              c*_ij = sgn(R_ij)
          else
              c*_ij = 0
          end if
      end for
  end for
```

The input data has G genes, M miRNAs and N samples including both tumor and nontumor tissues. The value of c_{ij} equals to 1 if the ith gene is a potential target of the jth miRNA based on sequence analysis, and 0 otherwise. Similarly, nonzero output values of c^*_{ij} indicate targeting relationships. Additionally, the sign of output values indicates correlation types: $+1$ for positive correlation and -1 for negative correlation.
