# Supplementary material for: iSubgraph: Integrative Genomics for Subgroup Discovery in Hepatocellular Carcinoma Using Graph Mining and Mixture Models
Source: PLoS One. 2013 Nov 4;8(11):e78624. doi: 10.1371/journal.pone.0078624 (PMC3817163; doi:10.1371/journal.pone.0078624)
Supplement: Procedure S2 — Patient Graph Construction Method. (PDF) [file pone.0078624.s002.pdf]

Procedure S2 Patient Graph Construction Method

Input: Gene expression data, $\{x_{ni}\}$,
miRNA expression data, $\{y_{nj}\}$,
correlated target predictions $\{c^*_{ij}\}$,
tag thresholds, $\{T_{x,i}\}, \{T_{y,j}\}$.

Output: Set of patient graphs, $\mathcal{GS}$.

```
𝒢𝒮 ← ∅
L ← {UP, DOWN}
for n ← 1 to N do
    V_n ← ∅, E_n ← ∅, ℓ_n ← ∅
    ▷ Insert graph nodes and assign tags
    for i ← 1 to G do
        if x_ni > T^+_{x,i} then
            V_n ← V_n ∪ {v^x_i}
            ℓ_n ← ℓ_n ∪ {v^x_i ↦ UP}
        else if x_ni < T^-_{x,i} then
            V_n ← V_n ∪ {v^x_i}
            ℓ_n ← ℓ_n ∪ {v^x_i ↦ DOWN}
        end if
    end for
    for j ← 1 to M do
        if y_nj > T^+_{y,j} then
            V_n ← V_n ∪ {v^y_j}
            ℓ_n ← ℓ_n ∪ {v^y_j ↦ UP}
        else if y_nj < T^-_{y,j} then
            V_n ← V_n ∪ {v^y_j}
            ℓ_n ← ℓ_n ∪ {v^y_j ↦ DOWN}
        end if
    end for
    ▷ Insert edges
    for each pair (v^x_i, v^y_j) s.t. v^x_i, v^y_j ∈ V_n do
        if (c*_ij = +1 and ℓ_n(v^x_i) = ℓ_n(v^y_j)) or
          (c*_ij = −1 and ℓ_n(v^x_i) ≠ ℓ_n(v^y_j)) then
            E_n ← E_n ∪ {(v^x_i, v^y_j)}
            ℓ_n ← ℓ_n ∪ {(v^x_i, v^y_j) ↦ ∅}
        end if
    end for
    𝒢𝒮 ← 𝒢𝒮 ∪ {(V_n, E_n, L, ℓ_n)}
end for
```

The input data has G genes, M miRNAs and N samples including only tumor tissues. The labeling functions $\ell.$ are defined as set of mapping rules. The parameter/variable types are denoted by the indices x and y for genes or miRNAs, respectively. The superscripts $+$ and $-$ indicate the thresholds for UP and DOWN tags, respectively.
