# Supplementary figures and images for: iSubgraph: Integrative Genomics for Subgroup Discovery in Hepatocellular Carcinoma Using Graph Mining and Mixture Models
Source: PLoS One. 2013 Nov 4;8(11):e78624. doi: 10.1371/journal.pone.0078624 (PMC3817163; doi:10.1371/journal.pone.0078624)

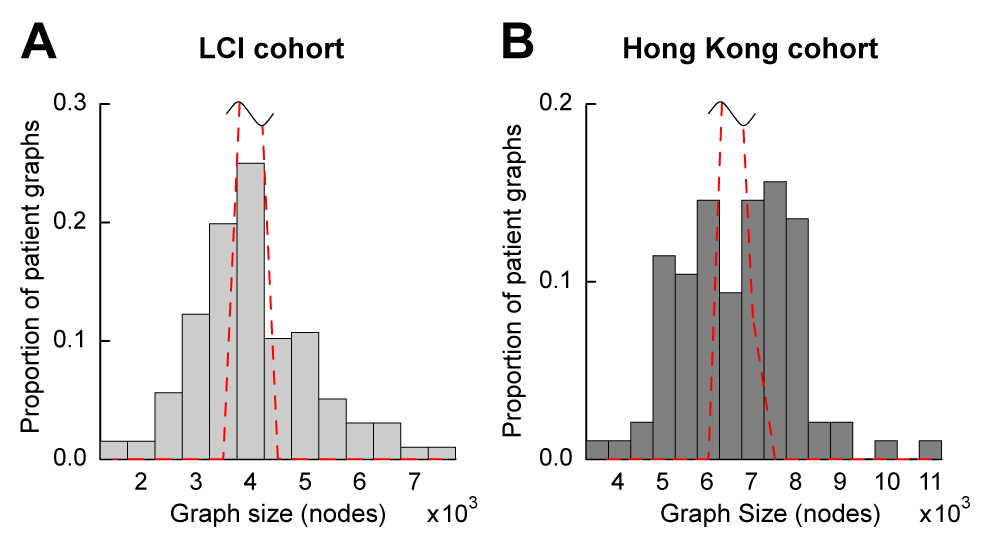

Supplement: Figure S1 — Histograms of patient graphs. (A) Histograms of patient graphs from the LCI () and (B) Hong Kong cohort () with respect to graph size. The red dashed curves, which are cut due to visualization purposes, show expected values. (TIF) [file pone.0078624.s003.tif]

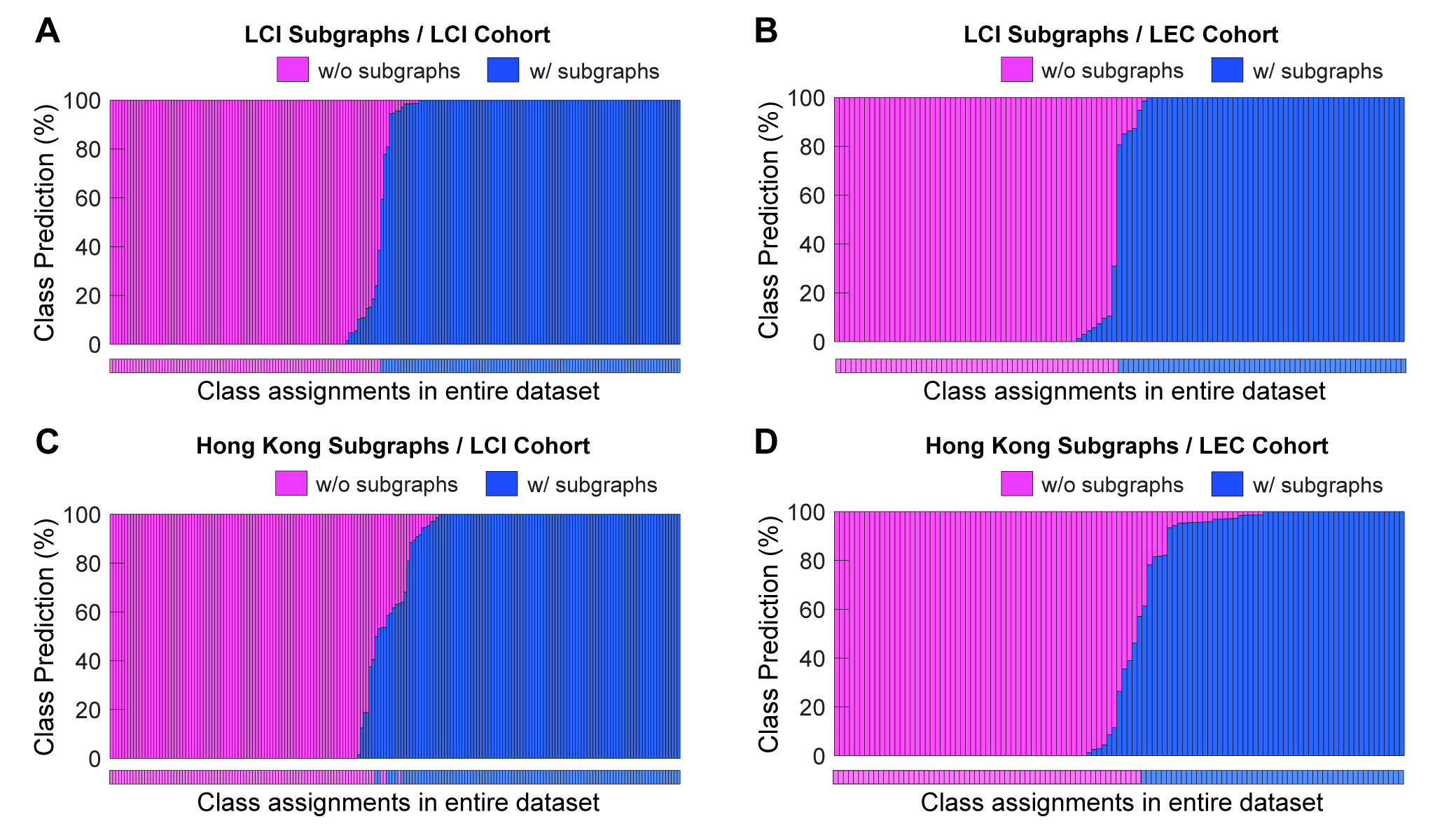

Supplement: Figure S2 — Robustness of class predictions. The proportion for each sample was calculated based on bootstrap prediction analysis (by resampling 70% of patients 100 times). The order of patients (-axis) is arranged according to proportions of class prediction in each panel. (A) Panels on the left show prediction analysis of the LCI cohort for the subgraphs from the LCI cohort and (C) Hong Kong cohort. (B) Panels on right show prediction analysis on the LEC cohort for the subgraphs from the LCI and (D) Hong Kong cohorts. Class assignments by the mixture models trained on entire dataset are shown below each plot. (TIF) [file pone.0078624.s004.tif]

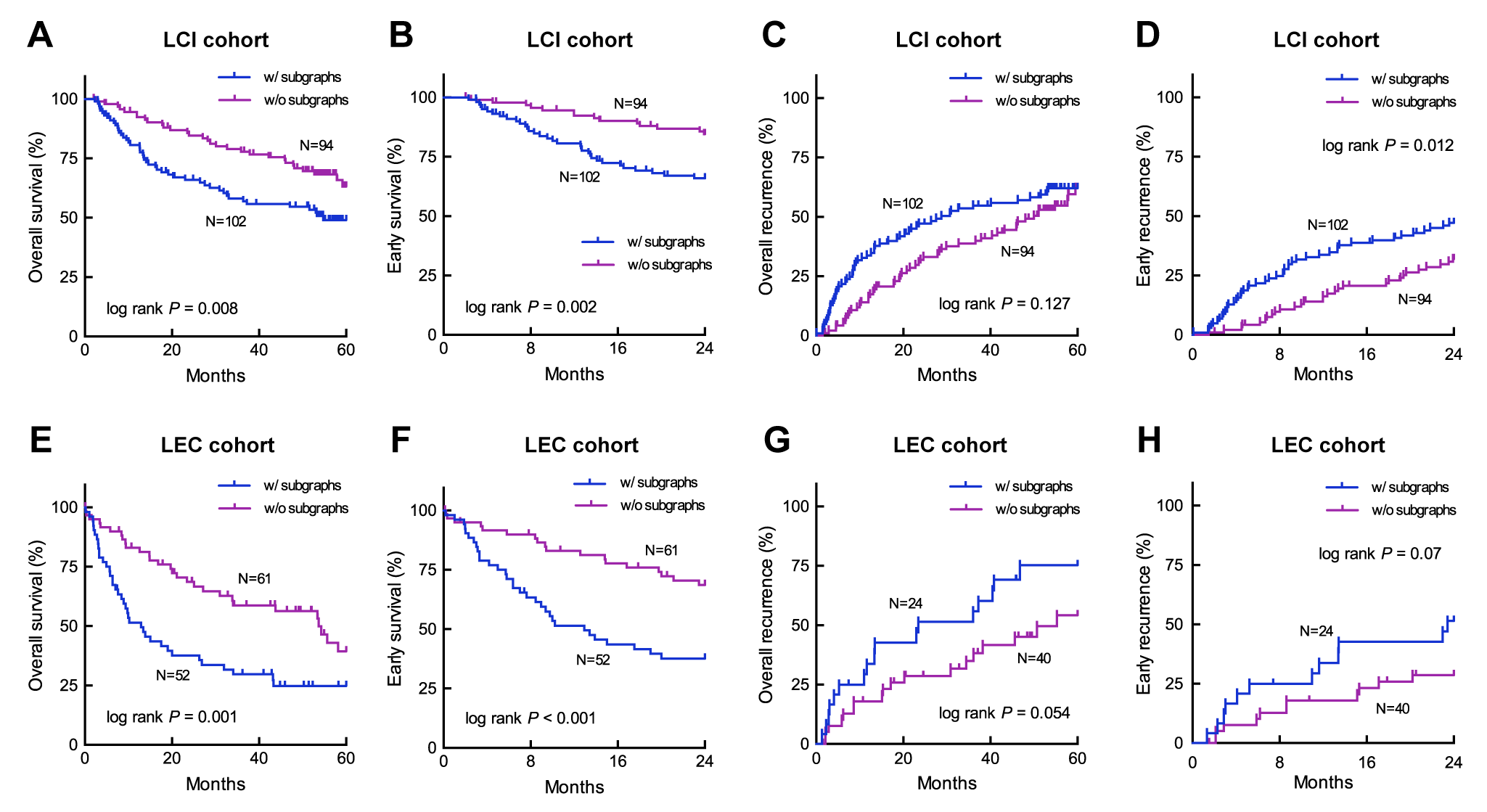

Supplement: Figure S3 — Combined Kaplan-Meier curves of patients subgrouped using the Hong Kong subgraphs. The first row shows the survival and recurrence characteristics of the LCI cohort () and the second row shows those of the LEC cohort (). The recurrence information was not available for some patients of the LEC cohort. From left to right, the columns indicate the overall survival (survival rates in the first 5 years), early survival (survival rates in the first 2 years), overall recurrence (disease-free survival rates in the first 5 years), and early recurrence (disease-free survival rates in the first 2 years) curves. -values were calculated by the Log-rank (Mantel-Cox) test. (TIF) [file pone.0078624.s005.tif]

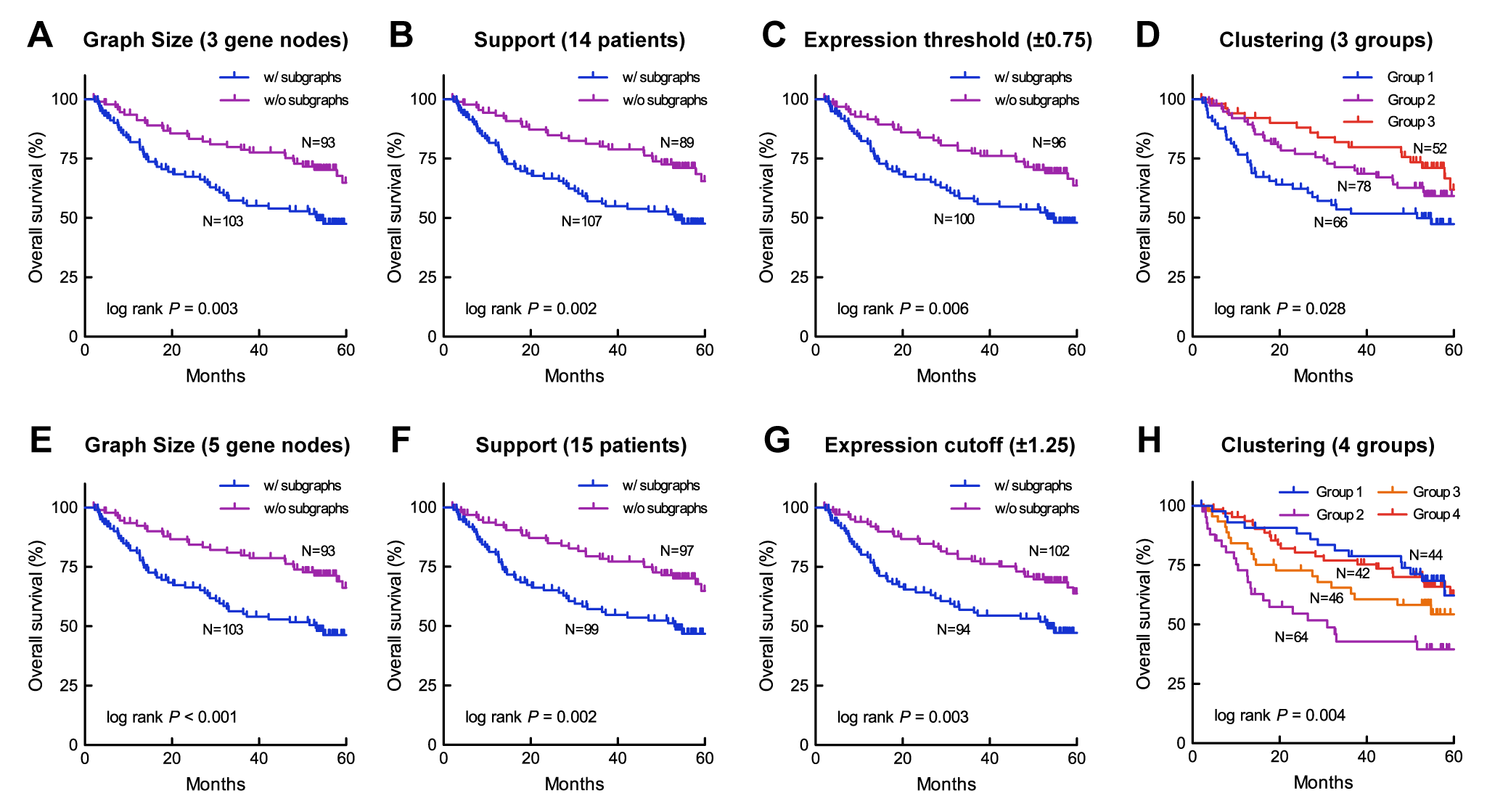

Supplement: Figure S4 — Kaplan-Meier curves of the LCI cohort subgrouped with small parameter perturbations. Survival curves in the first 5 years are shown for the LCI cohort () subgrouped by the mixture model. The parameter setting in Figure 9A is used as a reference, where -score cutoffs, minimum gene node count, support threshold and number of subgroups were set to , respectively. The experiments were repeated with perturbations in each panel, namely (A) decreasing the minimum gene node count to 3, (B) increasing the support threshold to 14, (C) decreasing the -score thresholds to and increasing the support threshold to 21, (D) increasing the number of subgroups to 3, (E) increasing the minimum gene node count to 5, (F) increase the support threshold to 15, (G) increasing the -score thresholds to and decreasing the support threshold to 8, (H) increasing the number of subgroups to 4. -values were calculated by the Log-rank (Mantel-Cox) test. (TIF) [file pone.0078624.s006.tif]

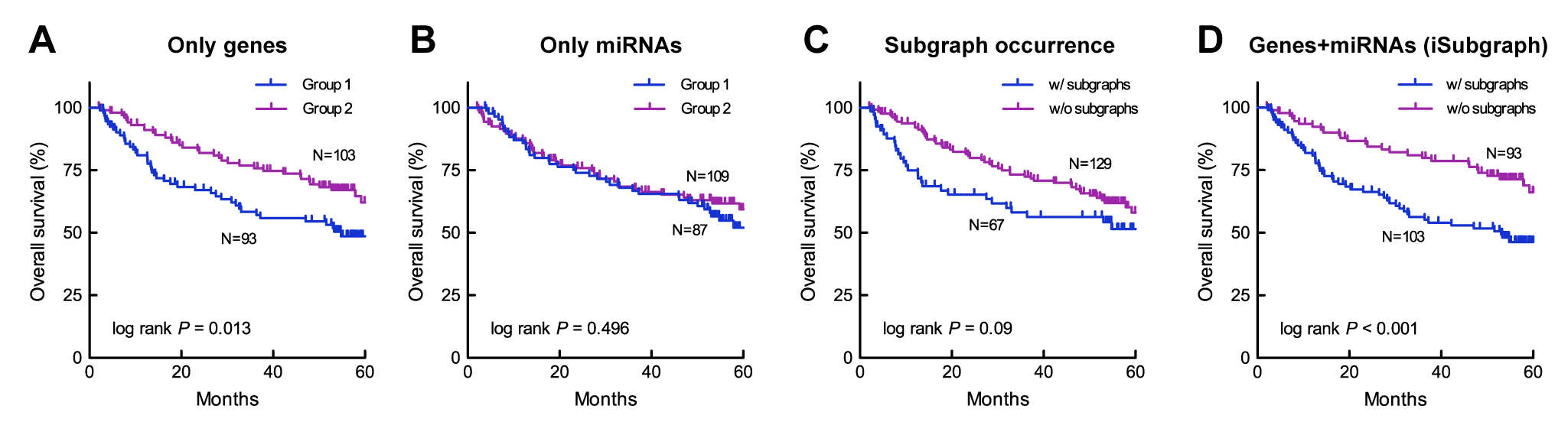

Supplement: Figure S5 — Kaplan-Meier curves of the LCI cohort subgrouped using different data types. Survival curves in the first 5 years are shown for the LCI cohort () subgrouped by the mixture model trained on (A) expression data of genes only, (B) expression data of miRNAs only, (C) occurrence data of the LCI subgraphs, and (D) expression data of genes and miRNAs found in the LCI subgraphs. -values were calculated by the Log-rank (Mantel-Cox) test. (TIF) [file pone.0078624.s007.tif]
