# Supplementary material for: iSubgraph: Integrative Genomics for Subgroup Discovery in Hepatocellular Carcinoma Using Graph Mining and Mixture Models
Source: PLoS One. 2013 Nov 4;8(11):e78624. doi: 10.1371/journal.pone.0078624 (PMC3817163; doi:10.1371/journal.pone.0078624)
Supplement: Table S1 — The number of closed frequent subgraphs and the number of genes and miRNAs in those subgraphs for different parameter settings in the LCI dataset. (PDF) [file pone.0078624.s008.pdf]

Table S1. The number of closed frequent subgraphs and the number of genes and miRNAs in those subgraphs for different parameter settings in the LCI dataset.

Parameter Set	#Subgraphs	#genes	#miRNAs
$(\pm 1, 13, 4, 2)$	6,280	384	49
$(\pm 1, 13, 3, 2)$	25,467	769	76
$(\pm 1, 13, 5, 2)$	876	183	28
$(\pm 1, 14, 4, 2)$	1364	219	33
$(\pm 1, 15, 4, 2)$	249	130	21
$(\pm 0.75, 21, 4, 2)$	4983	183	17
$(\pm 1.25, 8, 4, 2)$	685	93	9

The parameter sets include z-score cutoffs, minimum gene node count, support threshold and number of subgroups, respectively.
